# Stability of the hybrid epithelial/mesenchymal phenotype

**DOI:** 10.18632/oncotarget.8166

**Published:** 2016-03-17

**Authors:** Mohit Kumar Jolly, Satyendra C. Tripathi, Dongya Jia, Steven M. Mooney, Muge Celiktas, Samir M. Hanash, Sendurai A. Mani, Kenneth J. Pienta, Eshel Ben-Jacob, Herbert Levine

**Affiliations:** ^1^ Center for Theoretical Biological Physics, Rice University, Houston, TX, USA; ^2^ Department of Bioengineering, Rice University, Houston, TX, USA; ^3^ Department of Physics and Astronomy, Rice University, Houston, TX, USA; ^4^ Department of Biosciences, Rice University, Houston, TX, USA; ^5^ Graduate Program in Systems, Synthetic and Physical Biology, Rice University, Houston, TX, USA; ^6^ School of Physics and Astronomy and The Sagol School of Neuroscience, Tel-Aviv University, Tel-Aviv, Israel; ^7^ Department of Biology, University of Waterloo, Waterloo, ON, Canada; ^8^ Department of Clinical Cancer Prevention, University of Texas MD Anderson Cancer Center, Houston, TX, USA; ^9^ Department of Translational Molecular Pathology, University of Texas MD Anderson Cancer Center, Houston, TX, USA; ^10^ Red and Charline McCombs Institute for the Early Detection and Treatment of Cancer, University of Texas MD Anderson Cancer Center, Houston, TX, USA; ^11^ Metastasis Research Center, University of Texas MD Anderson Cancer Center, Houston, TX, USA; ^12^ The James Brady Urological Institute, and Departments of Urology, Oncology, Pharmacology and Molecular Sciences, Johns Hopkins School of Medicine, Baltimore, MD, USA

**Keywords:** partial EMT, epithelial-mesenchymal transition, cancer stem cells, multistability, cell-fate decisions

## Abstract

Epithelial-to-Mesenchymal Transition (EMT) and its reverse – Mesenchymal to Epithelial Transition (MET) – are hallmarks of cellular plasticity during embryonic development and cancer metastasis. During EMT, epithelial cells lose cell-cell adhesion and gain migratory and invasive traits either partially or completely, leading to a hybrid epithelial/mesenchymal (hybrid E/M) or a mesenchymal phenotype respectively. Mesenchymal cells move individually, but hybrid E/M cells migrate collectively as observed during gastrulation, wound healing, and the formation of tumor clusters detected as Circulating Tumor Cells (CTCs). Typically, the hybrid E/M phenotype has largely been tacitly assumed to be transient and ‘metastable’. Here, we identify certain ‘phenotypic stability factors’ (PSFs) such as GRHL2 that couple to the core EMT decision-making circuit (miR-200/ZEB) and stabilize hybrid E/M phenotype. Further, we show that H1975 lung cancer cells can display a stable hybrid E/M phenotype and migrate collectively, a behavior that is impaired by knockdown of GRHL2 and another previously identified PSF - OVOL. In addition, our computational model predicts that GRHL2 can also associate hybrid E/M phenotype with high tumor-initiating potential, a prediction strengthened by the observation that the higher levels of these PSFs may be predictive of poor patient outcome. Finally, based on these specific examples, we deduce certain network motifs that can stabilize the hybrid E/M phenotype. Our results suggest that partial EMT, i.e. a hybrid E/M phenotype, need not be ‘metastable’, and strengthen the emerging notion that partial EMT, but not necessarily a complete EMT, is associated with aggressive tumor progression.

## INTRODUCTION

During metastasis, cancer cells leave the primary tumor, travel through the circulation, and seed secondary tumors in distant organs. Metastasis can be fueled by the engines of cellular plasticity - bidirectional transitions between the epithelial and mesenchymal phenotypes - the Epithelial to Mesenchymal Transition (EMT) and its reverse the Mesenchymal to Epithelial Transition (MET) [1]. EMT and MET are not specific to cancer metastasis, rather they are fundamental developmental phenomena that get aberrantly activated in cancer metastasis [1]. Cells undergoing EMT lose their cell-cell adhesion and gain migratory and invasive traits to invade the basement membrane and enter the blood vessels as Circulating Tumor Cells (CTCs) [2]. These CTCs exit at distant organs and usually undergo MET to settle down and proliferate in order to form secondary tumors during metastasis [1]. Elucidating the principles of this cellular plasticity is expected to offer crucial clues for halting metastatic progression.

While transitioning between the E and M phenotypes, cells can adopt a hybrid epithelial/mesenchymal (E/M) or a partial EMT phenotype. Cells in this phenotype have a mix of epithelial (e.g. cell-cell adhesion) and mesenchymal (e.g. migration) traits that enable them to move collectively during mammary gland formation, trachea development, and wound healing [3, 4]. Such collective migration obviates the need for all cells to identify and respond to an external signal, and allows maximum cellular plasticity [3]. Furthermore, during metastasis, collective migration as CTC clusters has observed in the patients with lung, prostate, and breast cancer [5, 6]. Cells in CTC clusters can enter and exit the bloodstream more efficiently [7], are resistant to anoikis [6], and form up to 50-times more tumors as compared to CTCs that have undergone a complete EMT and move individually [6]. In addition, the implications of hybrid E/M phenotype in metastasis might help explain recent studies [8, 9] showing that preventing cells from switching to being fully mesenchymal does not drastically affect metastasis [10]. Furthermore, the predominance of cells co-expressing E and M markers in many aggressive tumors such as melanomas and basal-like and claudin-low breast cancers argues for a strong association of the hybrid E/M phenotype with aggressiveness [5]. Such co-expression, as compared to the expression of M genes only, correlates with increased invasive and metastatic potential and predicts poor outcomes independent of the breast cancer subtype [11]. Thus, the hybrid E/M phenotype can pose a higher metastatic risk in patients as compared to the pure M, i.e. complete EMT phenotype [5, 11].

Despite its critical significance in both physiological and pathological EMT, the hybrid E/M phenotype has not been comprehensively characterized. It has been proposed to be a ‘metastable’ phenotype (i.e. the cells cannot maintain this phenotype stably for a long time) that can be acquired only transiently *en route* to EMT [12, 13]. But recent experiments in mammary gland development show that the cells in terminal end buds (TEBs) can be maintained in a hybrid E/M phenotype and prevented from undergoing a complete EMT by transcription factor OVOL [14]. OVOL, thus, acts as a ‘phenotypic stability factor’ (PSF) [15] as proposed by recent attempts to mathematically model its role in mediating EMT/MET [16, 17]. Furthermore, OVOL not only stabilizes the hybrid E/M phenotype, but also has been predicted by mathematical models to enable the hybrid E/M cells to gain stemness [18], i.e. regenerative and self-renewal potential during developmental EMT, and the potential to seed secondary tumors during metastasis. These observations provide the motivation to identify more such ‘phenotypic stability factors’[15].

Here, *via* mathematical modeling, we identify two additional phenotypic stability factors that can expand the existence of the hybrid E/M phenotype - transcription factor GRHL2 and microRNA miR-145. Further, we show experimentally that partial EMT phenotype can be observed stably at a single-cell level in H1975 lung cancer cells *in vitro*, and knockdown of OVOL and GRHL2 can impair collective cell migration - a hallmark of the hybrid E/M phenotype - and drive a complete EMT. Finally, generalizing these findings by using a network topology-based approach, we propose several network motifs that can be utilized to identify the molecular players that can maintain a hybrid E/M phenotype and potentially are also likely to ascribe an enhanced tumor-initiating potential to the hybrid E/M phenotype.

## RESULTS

### Partial EMT can be stably maintained at a single-cell level *in vitro*

**Figure 1:**
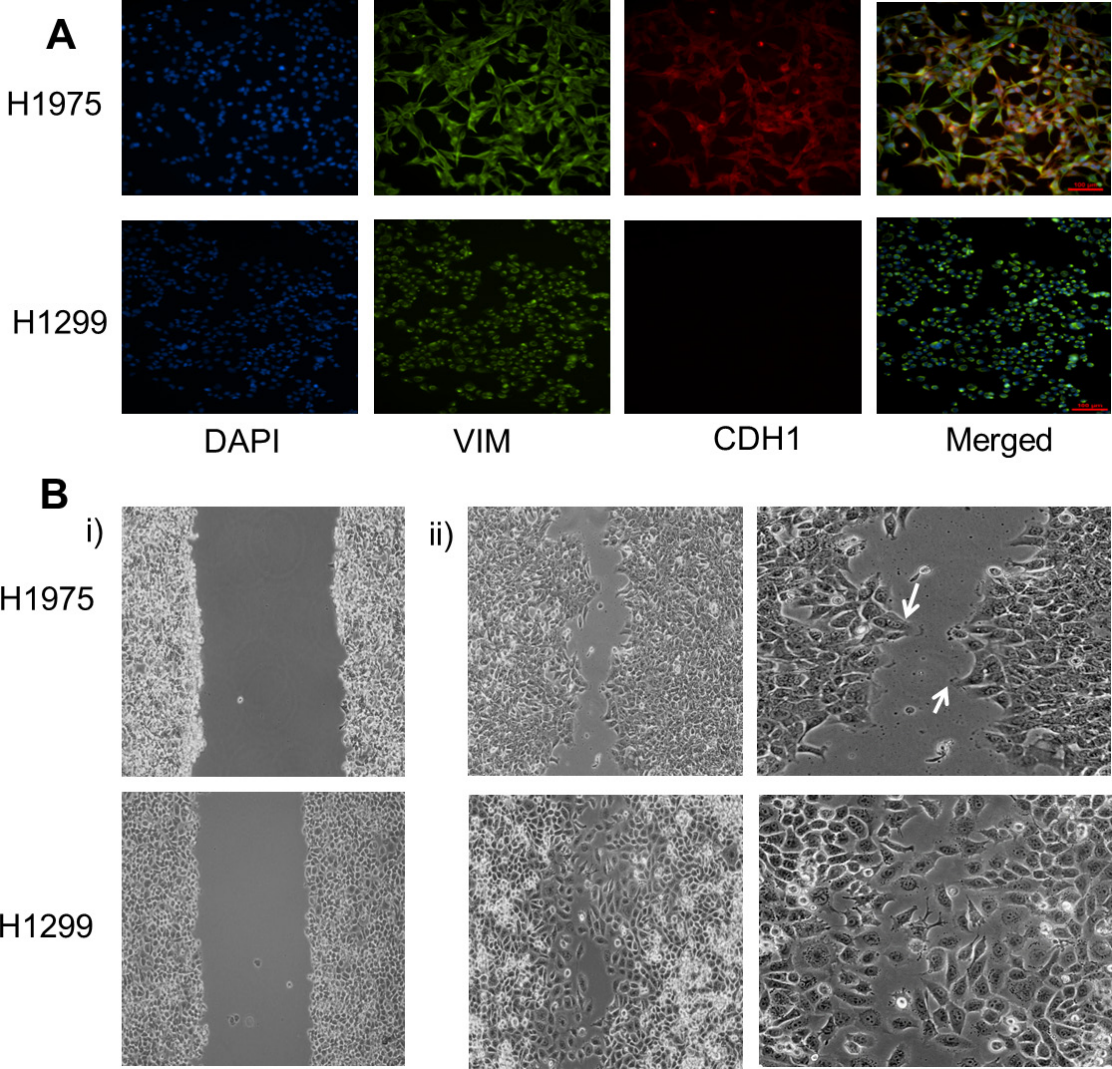
Characterizing the partial EMT phenotype **A.** Expression of CDH1 (E-cadherin) and VIM (Vimentin) examined by immunofluorescence staining. Scale bar 100 μm. **B.** Scratch assay of H1975 and H1299 depicting different cell motility patterns. White arrows denote finger-like projections seen in H1975 cells. Panel i) shows the condition at the beginning of scratch assay (*t* = 0 hours) whereas ii) shows for *t* = 12 hours for H1299 and *t* = 16 hours for H1975 cells.

We characterized three lung adenocarcinoma cell lines H1299, H2291, and H1975 for their EMT status by immunofluorescence (IF) staining with canonical EMT markers - CDH1 (E-cadherin) and VIM (Vimentin). H1299, categorized as a mesenchymal cell line [19], stained only for Vimentin; however, H2291 and H1975, both categorized as E/M cell lines based on ensemble measurements [19], stained for both VIM and CDH1. H2291 contained subpopulations of E (cells staining only for CDH1) and M (cells staining only for VIM) (Figure S3A), but H1975 cells co-stained for both VIM and CDH1 individually (Figure 1A), indicating a hybrid E/M or partial EMT phenotype at a single-cell level. Similar co-expression of E and M markers at a single-cell level has been observed in multiple cell lines belonging to breast cancer [11, 17] and lung cancer [20], as well as in metastatic brain tumors [21], CTCs(22), and tumor buds - clusters of 1-5 malignant cells observed at the invasive front of the tumor [23]. Importantly, H1975 cells maintained this partial EMT phenotype at a single-cell level for over two months in culture, thereby demonstrating a stable phenotype (Figure S4).

A partial EMT phenotype has been implicated in collective cell migration during embryonic development and wound healing [3, 4, 12]; therefore, we conducted a scratch assay for H1299 and H1975 cell lines. H1299 cells moved largely as single cells, but H1975 cells filled the wound moving collectively and forming finger-like projections (Figure 1B). These finger-like projections are the hallmarks of collective migration as noted earlier for migration of TEBs [14], and suggest that collective cell migration as sheets or clusters might be observed in tumors as well, as also reflected by tumor budding [23] and the migration of CTCs in clusters that are formed before entering the circulation [24].

### Mathematical modeling predicts GRHL2 and miR-145 act as ‘phenotypic stability factors’ for the partial EMT phenotype

**Figure 2:**
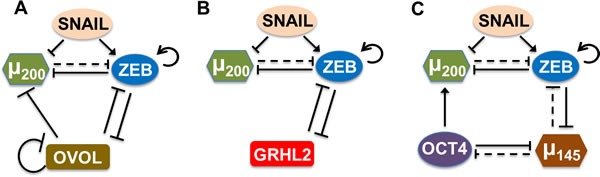
Coupling of core EMT circuit (miR-200/ZEB driven by SNAIL) with other ‘phenotypic stability factors’ (PSFs) **A.** OVOL forms a mutually inhibitory loop with ZEB, inhibits miR-200, and self-inhibits. **B.** GRHL2 forms a mutually inhibitory loop with ZEB. **C.** miR-145 and OCT4 mutually inhibit each other, miR-145 and ZEB also form a double negative loop, and OCT4 activates miR-200. Solid lines represent transcriptional regulation, while dotted lines represent miRNA-mediated regulation; arrows denote activation, bars for inhibition. Details of coupling for these PSFs with miR-200/ZEB are in SI section 1.

Previous experimental and theoretical analysis shows that the transcription factor OVOL can play a crucial role in maintaining a hybrid E/M phenotype [14, 16, 17]. Here, we investigate other similar ‘phenotypic stability factors’ (PSFs) that have been proposed to (a) maintain collective cell migration, or (b) induce a partial EMT, or (c) couple to the ‘motor of cellular plasticity’ - a double negative feedback loop between miR-200 and ZEB that regulates EMT/MET in multiple tumors [25] - in a similar manner as that of OVOL (Figure 2; SI sections 1,2). First, GRHL2 - a well-known regulator of morphogenesis that controls the differentiation and migration of epithelial cell layers - can maintain collective cell migration at its endogenous levels, drives MET when overexpressed, and enables EMT when knocked down in breast cancer cells [26, 27]. Second, miR-145 has been shown to induce a partial EMT in prostate cancer cells [28], and similar to OVOL and GRHL2, can drive MET when overexpressed [28]. All of these factors - OVOL, GRHL2, and miR-145 - couple to the (miR-200/ZEB) loop that has been proposed to act as a three-way switch - enabling three phenotypes - E (high miR-200,low ZEB), M (low miR-200, high ZEB), and hybrid E/M (medium miR-200, medium ZEB) [29].

As a first step towards elucidating the effect of these factors on the epithelial-hybrid-mesenchymal transition, we investigate their coupling with (miR-200/ZEB), and evaluate the response of the coupled circuits to different levels of SNAIL, an input to the (miR-200/ZEB) circuit, *via* a mathematical model that considers the dynamics of miR-200, ZEB mRNA, ZEB protein, and GRHL2 protein as a function of their regulatory interactions, and treats SNAIL as a driving input to this circuit.

**Figure 3:**
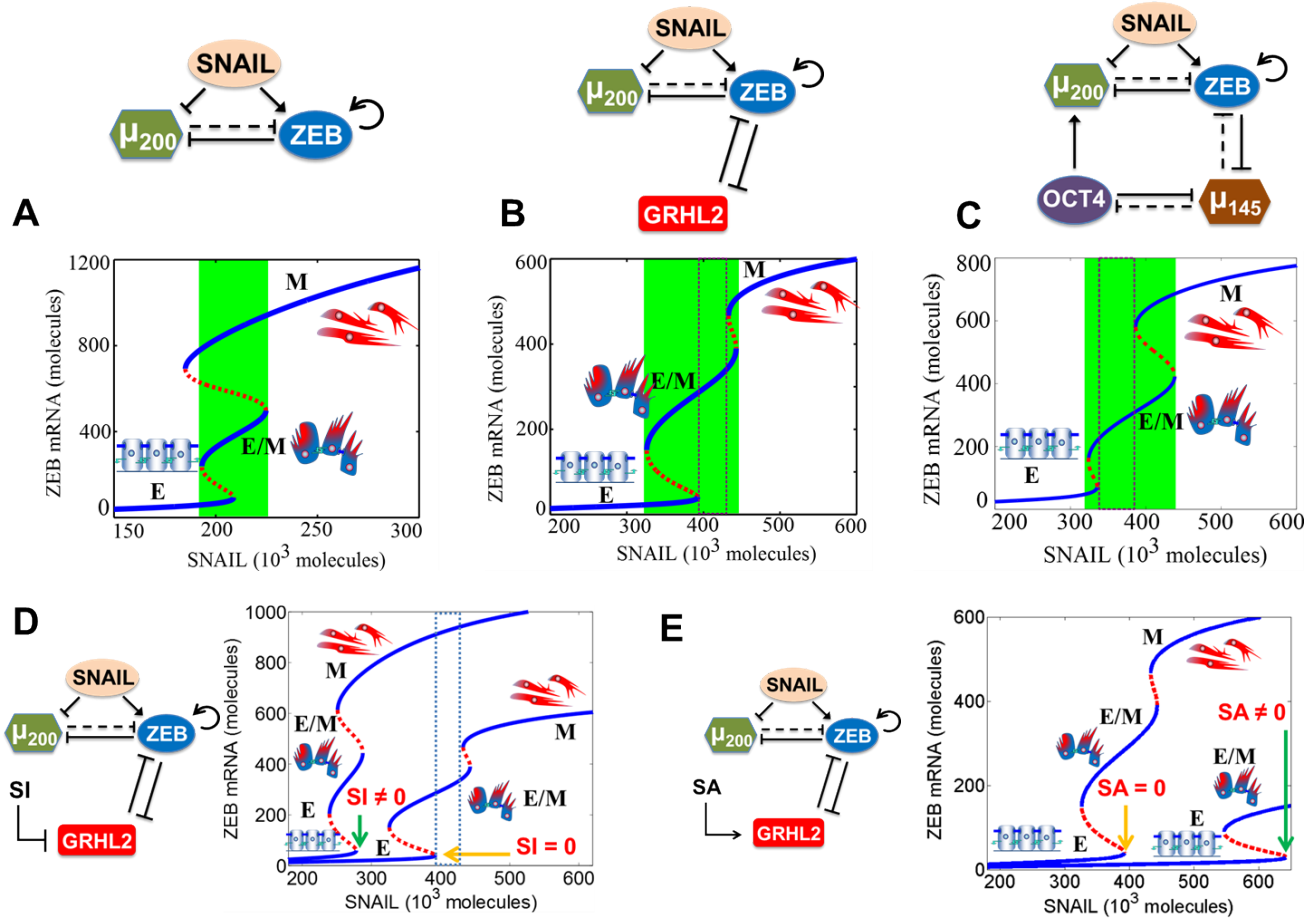
Dynamical system characteristics of miR-200/ZEB circuit when coupled with GRHL2 and miR-145/OCT4 separately Bifurcation of mRNA levels of ZEB in response to SNAIL levels for **A.** miR-200/ZEB circuit, **B.** miR-200/ZEB/GRHL2 circuit, **C.** miR-200/ZEB/miR-145/OCT4 circuit, **D.** miR-200/ZEB/GRHL2 circuit when GRHL2 is inhibited by external signal SI, and **E.** miR-200/ZEB /GRHL2 circuit when GRHL2 is activated by external signal SA. Note the x-axis range change from (A) to (B), (C), (D), and (E). The region marked by green in panels (A), (B), (C) represents the range of SNAIL levels for which the hybrid E/M phenotype can exist alone or as one of the multiple possible phenotypes; and that marked by dotted rectangle in (B), (C), and (D) represents the range of SNAIL levels for which the hybrid E/M phenotype can exist alone. Corresponding cartoons have been drawn alongside the phenotype. Blue solid lines denote stable steady states or phenotypes, and red dotted lines indicate unstable steady states. Yellow arrows in (D) and (E) indicate the SNAIL values at which cells can exit an epithelial phenotype for the control case (i.e. GRHL2 is not activated or repressed), whereas green arrows indicate the same for the case when GRHL2 is inhibited ((D)) or activated (E)).

The response of the different circuits to varying levels of SNAIL (mimicking the effect of EMT-inducing signals) is presented as a bifurcation diagram of ZEB mRNA levels in Figure 3. Lower levels (< 100 molecules) of ZEB mRNA denote an epithelial (E) phenotype, intermediate values (∼200-450 molecules) correspond to a hybrid E/M state and higher values denote a mesenchymal (M) phenotype (blue solid lines in Figure 3). For low SNAIL levels, cells attain only the E phenotype. With increase in SNAIL levels, EMT is induced partially hence cells attain a hybrid E/M phenotype, and further increases in SNAIL levels induce a complete EMT and consequently cells attain a mesenchymal (M) phenotype. The range of SNAIL values for which the hybrid E/M phenotype exists is much larger for the (miR-200/ZEB/GRHL2) circuit as compared to that for the (miR-200/ZEB) circuit (i.e. without being coupled with GRHL2) (compare the green area in Figure 3B *vs* that in Figure 3A). Most importantly, there is now a range for which the only possible state is partial EMT (region in the dotted rectangle), and therefore, in this range, the hybrid E/M phenotype is absolutely stable. In other words, after incorporating the effect of GRHL2, a parameter regime (physiological conditions) under which most cells will attain a stable hybrid E/M phenotype is enabled. Equivalently, if the cells present in the parameter region corresponding to the dotted region in Figure 3 will be sorted by flow cytometry (FACS), most of them will express both E and M markers, thereby corresponding to a hybrid E/M phenotype. A similar set of results is also observed for the (miR-200/ZEB/miR-145/OCT4) circuit (compare the green area in Figure 3C *vs* that in Figure 3A), thereby capturing the role of the ‘phenotypic stability factors’ (PSFs) GRHL2 and miR-145 in stabilizing the hybrid E/M phenotype.

Next, we investigated the effect of overexpression and inhibition of these ‘phenotypic stability factors’ by considering an external activating (SA) or inhibiting signal (SI) on GRHL2. Our model predicts that similar to the results obtained for OVOL [16], knockdown of GRHL2 and miR-145 destabilizes the hybrid E/M phenotype (note the absence of the region bound by dotted rectangle in case of SI ≠0 in Figure 3D) and can lead to a complete EMT(note the much lower levels of SNAIL needed to induce a partial or complete EMT when SI≠0 in Figure 3D), and that their overexpression can induce an MET (note the much higher level of SNAIL needed to induce a partial EMT when SA≠0 in Figure 3E) (also see Figure S5). These predictions are consistent with experiments showing that (a) knockdown of OVOL and GRHL2 impairs collective cell migration during mammary morphogenesis and lung development respectively [14, 27], (b) overexpression of GRHL2 and OVOL drives MET in MDA-MB-231 and PC3 cells respectively [26, 30], and (c) overexpression of miR-145 represses EMT in PC3 and DU145 cells *in vitro* [28], and the aggressiveness of PC3 cells in the bone *in vivo* [31].

### Experiments demonstrate that knockdown of OVOL and GRHL2 leads to a complete EMT

**Figure 4:**
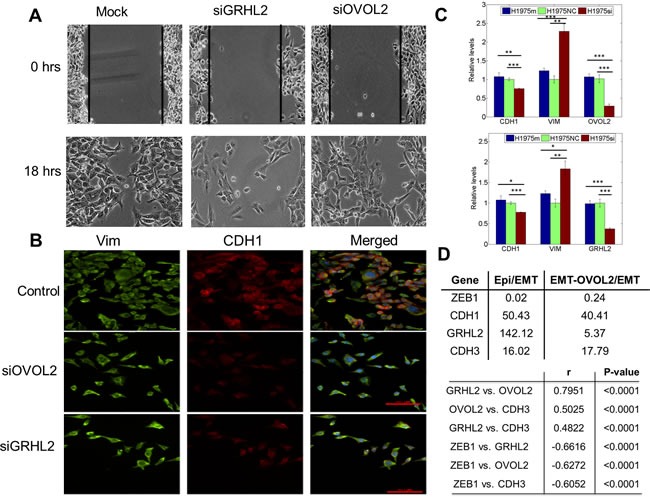
Knockdown of GRHL2 and OVOL2 in H1975 cells, and expression values of GRHL2, CDH3, and OVOL in different PC-3 clones **A.** Scratch assay of H1975 cells for the control case, and when treated with siRNA against GRHL2 and OVOL2, depicting different cell motility patterns. **B.** Expression of CDH1 (E-cadherin, red) and VIM (Vimentin, green) examined by immuno-fluorescence staining. Scale bar 100 μm. The blue color stains DAPI. **C.** Quantitative RT-PCR for CDH1, VIM, GRHL2, and OVOL2 after and before treatment with siRNAs against GRHL2 and OVOL2. Top panel is for siRNA against GRHL2, bottom one for that against OVOL2. H1975si shows the case when cells are treated with siRNA, H1975 NC denotes the negative control, and H1975m denotes mock case. *, *p* < = 0.05; **, *p* < 0.005; and ***, *p* < 0.0005. Error bars represent standard deviation, n = 3. **D.** (top) Fold-change in expression levels (log2) of GRHL2, OVOL2, CDH3, and ZEB1 in PC3-Epi *vs*. PC3-EMT, and PC3-EMT-OVOL2 *vs*. PC-EMT clonal cell lines. (bottom) Correlation analysis for GRHL2, OVOL2, CDH3, and ZEB1 expression in NCI-60. Pearson correlation coefficients (r) and *p*-values (two-tailed) are given.

To directly test our prediction that knockdown of these PSFs can drive a complete EMT, we investigated the effect of silencing OVOL2 and GRHL2 in H1975 cells, which exhibit a stable hybrid E/M phenotype, *via* siRNAs. We observed that on treatment with siRNAs against GRHL2 and OVOL2, the collective migration of H1975 cells was disrupted, and they migrated more individually, suggesting the completion of EMT (Figure 4A). Consistently, cells lost E-cad (CDH1) expression both at the transcriptional and the translational level (Figure 4B, 4C) and gained ZEB1 (Figure S6). Disruption of collective migration was more pronounced in cells treated with siGRHL2, possibly because of the crucial role of GRHL2 in lung development [27]. Knockdown of GRHL2 and OVOL2 also restricted cell proliferation (Figure S6) - another cellular trait usually associated with EMT.

Next, we examined the levels of GRHL2 and OVOL (OVOL1/2) in two stable clonal cell lines derived from PC3 - PC3-Epi (epithelial clonal population), and PC3-EMT (mesenchymal clonal population) [30, 32], and observed significant differences in the ratio of PC3-Epi/PC3-EMT levels for GRHL2 (142.12x), OVOL1 (174.43x), OVOL2 (25.89x), as well as ZEB1 (0.02x) and E-cadherin (50.43x) (Figure 4D, S7A), indicating that EMT or MET is associated with differential expression of these players. Overexpression of OVOL1 and OVOL2 in PC3-EMT cells can drive a MET [30]; but GRHL2 levels were not substantially upregulated (Figure 4D, S7A), indicating that GRHL2 and OVOL might be operating independent of each other in PC3 cells. However, the functional association between GRHL2 and OVOL might be tissue-specific, because GRHL2 can activate OVOL during nephric bud development [33] and trophoblast branching morphogenesis [34]. These contextual differences notwithstanding, GRHL2 and OVOL1/2 were recently demonstrated to be highly correlated with ‘NCI-60 consensus epithelial’ (NEC) signature [35] - a list of genes such as CDH1 (E-cadherin) that are involved in maintaining adherens and/or tight junctions.

### ‘Phenotypic stability factors’ may predict poor prognosis

Motivated by the correlation of GRHL2 and OVOL with the NEC signature, we investigated their levels in the NCI-60 cohort that has been classified as epithelial, mesenchymal, and hybrid E/M groups of cell lines based on their CDH1/VIM expression ratio [36]. ZEB1 (the EMT-inducing TF in our core EMT circuit), OVOL2, and CDH3 (P-cadherin; a proposed marker of hybrid E/M phenotype [37]; 16.02x in PC3-Epi as compared to PC3-EMT) had statistically significant different levels in the different categories (E, M, and E/M) of the NCI-60 cell lines (Figure S7B). Further, the levels of GRHL2, OVOL2, and CDH3 were positively correlated with one another; and all of them negatively correlated with ZEB1 (Figure 4D). The strongest association observed was between GRHL2 and OVOL2, endorsing our model predictions about similar roles of OVOL and GRHL2 in mediating epithelial plasticity. In addition, OVOL1/2 is highly correlated with GRHL2 and CDH3 (positively) and with ZEB1 (negatively) in a panel of 877 cell lines [30] from The Cancer Cell Line Encyclopedia (CCLE) [38].

**Figure 5:**
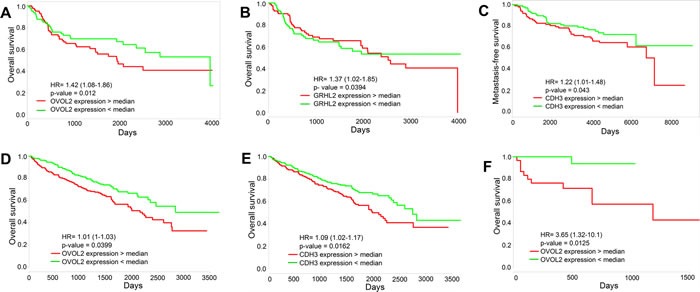
Survival analysis Overall survival and metastasis-free survival for the expression of GRHL2, OVOL2 and CDH3 individually in multiple tissue types - **A.**, **B.** GSE13507 (*n* = 164), **C.** GSE7390 (*n* = 197), **D.**, **E.** TCGA_KIRC (*n* = 505), **F.** TCGA_COAD (*n* = 120).

Finally, the levels of the PSFs OVOL2 and GRHL2, and the proposed marker for a hybrid E/M phenotype CDH3 can correlate with poor overall survival (OS), metastasis -free survival (MFS), and relapse-free survival (RFS) across multiple tissue types (Figure 5, S8). Their low levels, indicative of a completely mesenchymal phenotype (Figure S3B, C), associate with a better survival, thereby arguing that complete EMT need not be the hallmark of aggressiveness. On the other hand, cells in breast and lung carcinomas usually shed some epithelial traits to migrate, invade and metastasize [2]. Therefore, the relatively high levels of CDH3, OVOL2, and GRHL2 in patients with poor survival might highlight the role of hybrid E/M phenotype in metastatic progression, at least in these carcinomas. These survival curves are reminiscent of a recent study of 1678 independent human breast cancer samples indicating that a GRHL2-mediated gene-set pair can “effectively stratify patients showing significant differences in metastasis-free survival”[39], and are congruent with reports indicating that higher levels of GRHL2 correlate with shorter RFS in breast cancer patients [40], and with lower OS and RFS in colorectal cancer patients [41].

### Mathematical modeling predicts that GRHL2 can associate hybrid E/M phenotype with high tumor-initiation potential

Consistent with the emerging idea of the role of hybrid E/M phenotype in metastatic progression [5], hybrid E/M cells have also been recently shown to possess high tumor-initiating or stem-like properties, sometimes even higher than those possessed by cells with a complete EMT phenotype [11, 20]. In other words, the ‘stemness window’ (range of conditions under which cells attain stem-like traits) lies somewhere mid-way on an ‘EMT axis’ (with E and M as its two ends) [18, 42] as postulated earlier [43]. Here, we investigate how PSFs such as GRHL2 affect the positioning of the ‘stemness window’ on the ‘EMT axis’.

**Figure 6:**
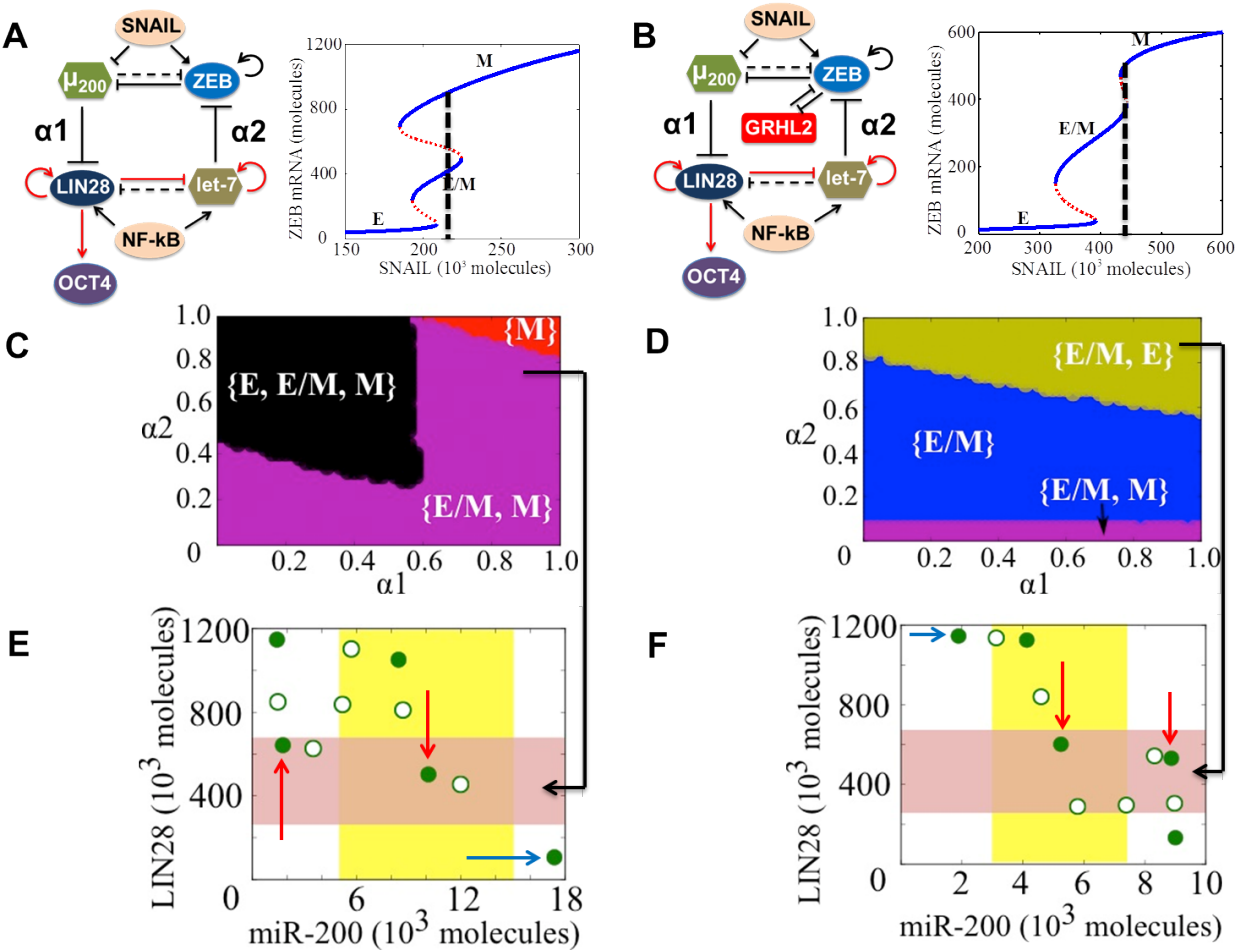
State-space characteristics of coupled networks miR-200/ZEB/LIN28/let-7 and miR-200/ZEB/LIN28/let-7/GRHL2, when cells are in {E/M, M} phase at α1 = α2 = 0 **A.**, **B.** miR-200/ZEB /LIN28/let-7 and miR-200/ZEB/GRHL2/LIN28/let-7 circuits respectively. Black solid lines represent transcriptional regulation; red lines denote translational self-activation of LIN28 (46) and activation of its own processing by the microRNA let-7 (47), and dotted lines denote miRNA-mediated regulation. The parameters α1 and α2 denote the strength of the ‘feed-forward coupling’ (miR-200 inhibiting LIN28) and ‘feed-backward coupling’ (let-7 inhibiting ZEB) respectively, and lie between 0 and 1. Larger values denote stronger inhibition. The dashed line in the bifurcation diagrams next to the circuits shows the phase in which cells are present when there is no coupling between EMT and stemness circuits (α1 = α2 = 0). Steady state diagram and the phase diagram in every column are for the circuit drawn in the topmost row of that column. **C.** Phase diagram of the circuit miR-200/ZEB/LIN28/let-7 representing the values of (α1, α2) for which the different phenotypes can lie in the stemness window, for SNAIL = 220*10^3^ molecules and NF-kB = 25*10^3^ molecules. **E.** Phenotypic map of the coupled circuit at α1 = α2 = 0.8 and at driving signals SNAIL = 220*10^3^ molecules and NF-kB = 25*10^3^ molecules. Red shaded area shows the ‘stemness window’ based on relative OCT4 levels, and yellow shaded area represents the range of miR-200 levels for the existence of the hybrid E/M phenotype, as noted in (29) for (miR-200/ZEB) circuit and in SI section 10 for (miR-200/ZEB/GRHL2) circuit. **D.**, **F.** represent a similar case for (C), (E) respectively but for the circuit with GRHL2, therefore SNAIL = 440*10^3^ molecules. Different colors represent different combinations of phenotypes that can gain stemness. The red arrows highlight the phenotypes that lie in the ‘stemness window’, and blue arrows denote some cases where the phenotypes lie outside of the ‘stemness window’. Green filled circles denote the stable steady states, and green hollow circles show the unstable steady states of the coupled circuits as denoted in (A) and (B).

The EMT-stemness interplay is regulated by coupling between the core EMT module (miR-200/ZEB) [25, 29] and the core stemness module (LIN28/let-7) [44–47]. They couple to each other *via* two links - miR-200 inhibits LIN28 [48] (hereafter called ‘feed-forward coupling’) and let-7 inhibits ZEB *via* HMGA2 [49, 50] (hereafter called ‘feed-backward coupling’) (Figure 6A, 6B). The strengths of these links are represented by α1 and α2 respectively. Both α1 and α2 are defined to be between 0 and 1; the larger their values, the stronger the corresponding inhibition. It may be noted that we are ignoring the feedback from OCT4 to miR-200 in our current analysis. Previous experimental and theoretical analysis has indicated that cells are most likely to be stem-like at intermediate levels of OCT4 (a downstream target of LIN28); both very high and very low levels of OCT4 cause the cells to differentiate (51-55). Taking this clue, we choose a ‘stemness window’, i.e. the representative range of levels of OCT4 for which the cells have the maximum likelihood to gain stemness to be (0.25-0.65) relative to the saturation level of OCT4 when it is activated by the maximum levels of LIN28 (marked by the red shaded region in Figure 6E, 6F). Notably, this range of OCT4 levels chosen for the ‘stemness window’ is likely to be context-specific; the results shown here are for a specific range to illustrate the concept.

To investigate the effect of GRHL2 on the EMT-stemness interplay, we calculate the steady states of the miR-200/ZEB/LIN28/let-7 and miR-200/ZEB/LIN28/let-7/GRHL2 circuits - both driven by SNAIL - at different values of (α1, α2), and plot the ‘stemness region’, i.e. range of (α1, α2) values for which these phenotypes - E, E/M, and M - can gain stemness as measured by levels of OCT4. We start with high levels of SNAIL such that at no coupling (α1 = α2 = 0), all cells are in either a mesenchymal or hybrid E/M phenotype and possess stem-like traits (phase {E/M, M} at α1 = α2 = 0 in Figure 6A). In the absence of GRHL2, a large range of values of (α1, α2) allow any of the three phenotypes (E, M, and E/M) to gain stemness, thereby reflecting an enriched flexibility of the ‘stemness window’ on the ‘EMT axis’. However, upon including GRHL2, this tristable phase {E, E/M, M} disappears (compare the black area in Figure 6D *vs*. the absence of that in Figure 6C). Furthermore, the miR-200/ZEB/LIN28/let-7/GRHL2 circuit enables, for a significant range of the values of (α1, α2), an exclusive association of the hybrid E/M phenotype with stemness, an association not observed in the miR-200/ZEB/LIN28/let-7 circuit (compare the blue area in Figure 6D *vs*. the absence of that in Figure 6C). Also, at strong bidirectional coupling (α1 = α2 = 0.8), in the absence of GRHL2, both E/M and M phenotypes can attain stemness (cells can also attain an E phenotype, but E phenotype lies outside the ‘stemness window’) as shown by blue arrow in (Figure 6E), whereas in presence of GRHL2,both E and E/M phenotypes can gain stemness but the M phenotype is precluded (compare the green region in Figure 6D *vs*. the absence of that in Figure 6C; and the states lying in ‘stemness window’ in Figure 6F *vs*. those in Figure 6E). The cells can still attain M phenotype, but it lies outside the ‘stemness window’ (see blue arrow in Figure 6F). The stem-like features of both E and E/M phenotypes are reminiscent of experiments showing two sub-populations of Cancer Stem Cells (CSCs) - one more E-like, and the other commensurate with a hybrid E/M phenotype [56].

Next, we choose a different value of SNAIL such that at α1 = α2 = 0, all cells are mesenchymal ({M} phase) and can gain stemness. In the absence of GRHL2, only the M phenotype can gain stemness for almost the entire range of (α1, α2) values; however, including GRHL2 enables a large range of the values of (α1, α2) in which only the hybrid E/M phenotype lies in the ‘stemness window’ (Figures S9, 10). Therefore, collectively, these results suggest GRHL2 excludes the association of mesenchymal (M) phenotype with stemness, and promotes the association of hybrid E/M phenotype with stem-like traits. In other words, GRHL2 maintains the stemness window closer to the hybrid E/M phenotype (mid-point of ‘EMT axis’). Collectively, these results illustrate that GRHL2 can not only stabilize the hybrid E/M phenotype, but also enhances the likelihood of the hybrid E/M cells to be ‘stem-like’ or gain stemness.

Although yet to be directly experimentally tested, these predictions are congruent with recent reports demonstrating that overexpression of GRHL2 can promote tumor growth and metastasis *in vivo* [40, 57], and offer a possible explanation for downregulation of GRHL2 suppressing tumor growth *in vivo* [57].

### Proposing several network motifs for identifying ‘phenotypic stability factors’

Intrigued by the similar role played by OVOL, GRHL2, and miR-145 in stabilizing the hybrid E/M phenotype, we analyzed the similarities and differences in these circuits - (miR-200/ZEB/OVOL), (miR-200/ZEB/GRHL2), and (miR-200/ZEB/miR-145/OCT4). For ease of comparison, the (miR-200/ZEB/ miR-145/OCT4) circuit is effectively reduced to a (miR-200/ZEB/miR-145) circuit such that similar to OVOL and GRHL2, miR-145 inhibits ZEB and is inhibited by ZEB relatively weakly(26,30,58-60) as compared to the inhibition of miR-200 by ZEB(25) (Figure S11).

**Figure 7:**
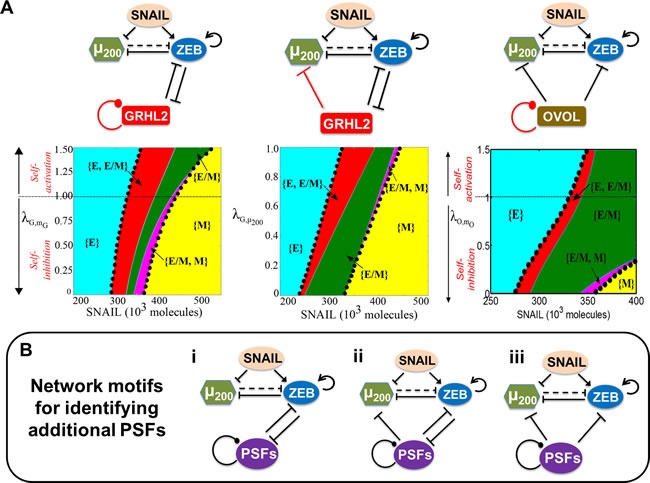
Network motifs that help maintain the hybrid E/M phenotype **A.** Top row denotes the different circuits that are investigated; the link whose strength is varied is depicted in red. Bottom row shows the phase-diagram of the miR-200/ZEB/GRHL2 circuit when driven by SNAIL and including a hypothetical self-regulatory link for GRHL ($\lambda_{G,m_{G}}$ denotes the strength of the self-regulation; $\lambda_{G,m_{G}}>1$ indicates self-activation, $\lambda_{G,m_{G}}<1$ implies self-inhibition, and $\lambda_{G,m_{G}}=1$ indicates no self-regulation) (left), phase diagram of the miR-200/ZEB/GRHL2 circuit when driven by SNAIL and a hypothetical inhibitory link from GRHL2 to miR-200 (the smaller the value of $\lambda_{G,\mu_{200}}$, the stronger the inhibition) (center), and phase diagram for the miR-200/ZEB/OVOL2 circuit when driven by SNAIL and including a hypothetical self-regulatory link for OVOL ($\lambda_{O,\mu_{O}}$ indicates the strength of self-regulation: $\lambda_{O,\mu_{O}}<1$ implies self-inhibition, $\lambda_{O,\mu_{O}}>1$ implies self-activation, and $\lambda_{O,\mu_{O}}=1$ shows no self-regulation) (right). In all these phase diagrams, the black dots bound the area of all phases that contain E/M as one of its phenotypes, and dark green region shows the phase when hybrid E/M phenotype need not co-exist with other phenotypes - {E/M} **B.** Proposed network motifs for identifying PSFs. The dot at the end of the self-regulatory link indicates that the PSF can potentially either self-activate or self-inhibit.

These three circuits have two key differences - (a) OVOL self-inhibits, miR-145 self-activates (in the effective circuit), and GRHL2 has no self-regulation (although it can self-activate in some biological contexts [27, 61]); and (b) OVOL and miR-145 (in the effective circuit) inhibit miR-200, but GRHL2 does not. Therefore, we investigate how two hypothetical links - self-regulation of GRHL2 and inhibition of miR-200 by GRHL2 - affect the range of parameters for the existence of E/M phenotype. We observe that the total area corresponding to the phases that contain E/M as one of the states or the only state increases monotonically as the strength of self-inhibition of GRHL2 is decreased and/or when the strength of self-activation of GRHL2 is increased (the area bounded by black dots in Figure 7A, left panel).

The other link - inhibition of miR-200 by GRHL2 - is represented by $\lambda_{G,\mu_{200}}$ (<1). We observe that the total area corresponding to phases containing E/M as one of the states does not change significantly by including an inhibitory link to miR-200 (the area bound by black dots in Figure 7A, middle panel). Therefore, neither the inhibitory link to miR-200 nor the self-regulatory action of the ‘phenotypic stability factor’ (PSF) qualitatively changes their behavior of stabilizing the hybrid E/M phenotype. Based on these results, we propose two network motifs to identify PSFs; if a particular transcription factor or miRNA couples with (miR-200/ZEB) in one of these two topologies, it is likely to act as a PSF for the hybrid E/M phenotype (Figure 7B, i-ii). In these motifs, a PSF forms a mutually inhibitory loop with ZEB, may inhibit miR-200, and may self-activate or self-inhibit (as shown by the dot at the end of the self-regulatory link) (Figure 7B, i-ii).

We also propose another network motif for identifying PSFs - a molecular player that can inhibit both miR-200 and ZEB (Figure 7B, iii). This motif is proposed by introducing the miR-200/ZEB/OVOL circuit where the inhibitory feedback from ZEB to OVOL is absent - as observed during mammary morphogenesis and epidermal development [14, 62] (Figure 7A, right panel). Again, relieving the self-inhibition and/or increasing the self-activation of OVOL only increases the total area corresponding to the phases that contain E/M as one of the phenotypes (the area bound by black dots in Figure 7A, right panel). Such an ‘incoherent’ external inhibitory signal that inhibits both miR-200 and ZEB can also increase the association of hybrid E/M phenotype with stemness (Figure S12–S14). Collectively, these three motifs can be utilized to identify additional factors that can stabilize a partial EMT.

Notably, the factors that form a mutually inhibitory loop with miR-200 instead of that with ZEB (Figure 7B, i-ii) cannot stabilize the hybrid E/M phenotype (Figure S15). Similar results are observed (Figure S15) when both miR-200 and ZEB are being activated instead of being inhibited (Figure 7B, iii), thereby illustrating the specificity of the proposed network motifs that can maintain a hybrid E/M phenotype. A different recent modeling attempt identified network motifs that can maintain a completely mesenchymal (M) or epithelial (E) phenotype [63, 64], but ours, to the best of our knowledge, is the first attempt to identify motifs that can stabilize a hybrid E/M or partial EMT phenotype.

## DISCUSSION

EMT and MET are fundamental processes during development, homeostasis, and diseases such as cancer and fibrosis that enable reversible bidirectional transitions between epithelial (E) and mesenchymal (M) phenotypes [2]. They have been largely considered to be binary or ‘all-or-none’ processes [1], but recently, a partial EMT or hybrid E/M phenotype has been increasingly recognized. This recognition is even more recent in EMT associated with cancer [17, 19, 22, 65–69] as compared to EMT associated with embryonic development and wound repair/tissue regeneration [4, 12, 70, 71]. This phenotype has been assumed to be a ‘metastable’ or transient state [12, 13] and CTCs have been observed to alter their EMT status dynamically [22], but recent experiments suggest a hybrid E/M phenotype can be stable [14, 72, 73]. Whether it is truly ‘metastable’ from a dynamical systems perspective [74] has been elusive.

The results here indicate that partial EMT need not be a ‘metastable’ transitory state attained *en route* to EMT, rather it can reflect a stable phenotype, especially in the presence of ‘phenotypic stability factors’ (PSFs) such as OVOL2, GRHL2, and miR-145. Experiments - including those shown here - showing that OVOL and GRHL2 knockdown can impair collective cell migration - the hallmark of partial EMT - and induce a complete EMT suggest that these PSFs can act as a “critical molecular brake on [a complete] EMT”[14]. Furthermore, GRHL2 can activate OVOL during nephric bud development and both of them induce the expression of epithelial effectors such as CDH1 [33], thereby forming a coherent feed-forward loop (FFL) [75], a common network motif that acts as a “stabilizer of target gene expression”[33]. The arrangement of two PSFs GRHL2 and OVOL in a FFL further strengthens the emerging notion that the hybrid E/M phenotype “defines [a] normal cell identity” and is not “necessarily [an] unstable transitory state”[76].

OVOL [18] and GRHL2 can not only stabilize a hybrid E/M phenotype, but also have been predicted to associate hybrid E/M phenotype with stemness, thereby suggesting a common design principle - stabilizing the hybrid E/M phenotype also potentially increases its likelihood to gain stemness While the role of these PSFs in maintaining the ‘stemness window’ around the midpoint of the ‘EMT axis’[18, 42] remains to be directly experimentally tested, the functional association of a hybrid E/M phenotype with stemness has been reported during EMT in developmental (type I EMT) [77], regenerative (type II EMT) [78], as well as pathological contexts (type III EMT) [79, 80]. Specifically, recent *in vitro* analysis of HMLER cells indicate that hybrid E/M cells form ten times more mammospheres as compared to a similar number of E and M cells [11]. Similar behavior of hybrid E/M cells was observed in multiple lung cancer cell lines [20]. Further, in ovarian and breast cancer metastasis, *in vivo* tumor growth is largely driven by hybrid E/M cells [81, 82]. Consistently, a transient activation, but not necessarily a prolonged activation, of EMT-inducing transcription factors including TWIST and SNAIL can significantly increase the tumor-initiating potential [83, 84]; suggesting that partial EMT is more stem-like than complete EMT.

The association of hybrid E/M phenotype with stemness [11, 45] is also strengthened by experiments indicating a link between the hybrid E/M phenotype and both *de novo* and acquired drug resistance [5]. Furthermore, the chemo-tolerant subpopulation in both basal-like and luminal breast cancer cell lines co-express both an epithelial marker CD24 and a mesenchymal marker CD44 [85], indicating its hybrid E/M status [11]. These CD24+ CD44+ cells also form more aggressive tumors *in vivo* as compared to the CD24-CD44+ (M) population [85]. In addition, a recent study demonstrated that co-treating the cells with EMT-inducing TGFβ and EMT-inhibiting Retinoic Acid (RA) [86] can enrich the hybrid E/M subpopulation (CD24+ CD44+) that is highly drug resistant [87].

Recent experiments indicate another possible PSF - ΔNp63α - that can induce a partial EMT in basal-like breast cancer cells by activating Slug (SNAIL2) as well as inhibiting ZEB *via* miR-205 [76]. Notably, CDH3 (P-cadherin) - a proposed marker of the hybrid E/M phenotype - is a downstream target of ΔNp63α [37]. The expression of ΔNp63α, Slug, and P-cadherin (CDH3) in myoepithelial cells [37, 76], and the activation of the p63 gene by GRHL2 in keratinocytes [61], further argues for the role of ΔNp63α in inducing a partial EMT. Whether ΔNp63α truly behaves as a PSF requires more careful analysis from a modeling perspective. For instance, the current model lumps together Snail1 and Snail2 (Slug) as SNAIL family, but they should be considered as two distinct entities to appreciate possible contextual differences between Snail1 and Slug - ΔNp63α activates Slug [76] but not necessarily SNAIL1, and Slug and Snail1 can have different roles in developmental and oncogenic EMT and may even inhibit each other [88, 89]. Not all PSFs need be expressed or be functionally active in the same context; also, their coupling with miR-200/ZEB might vary slightly in different tissues [90]. Therefore whether they act redundantly or synergistically is likely to be tissue-specific. Besides, the players that maintain a hybrid E/M state in fibrosis [91, 92] might be different from those reported here.

We also show two lung cancer cell lines classified as hybrid E/M based on population-based measurements - one of which predominantly contains hybrid E/M cells (H1975), and the other is largely an admixture of E and M cells (H2291). Importantly, H1975 cells can maintain their hybrid E/M phenotype for over two months in culture, and display collective migration, indicating their stable phenotype. Similar hybrid E/M cells co-expressing E and M markers have recently been reported in breast and lung cancer cell lines [11, 17, 20], yet most previous reports on partial EMT and those indicating EMT to be a spectrum of phenotypes are largely at an ensemble level [19, 64–66, 93, 94], thereby being inconclusive whether they contain admixtures of E and M cells, or individual hybrid E/M cells. Of course, these two manifestations of hybrid E/M - population level and single-cell level - need not be mutually exclusive, for instance, HMLER cells contain subpopulations for E, M and hybrid E/M cells [11].

How many intermediate states exist *en route* EMT, what are their different gene expression profiles, and what is their relative stability? These challenging questions remain unanswered, providing a fertile ground for integrating modeling and experimental approaches [17, 63, 64, 95, 96]. The direct coupling of the PSFs OVOL and GRHL2 to ZEB instead of SNAIL strengthens the claim that (miR-200/ZEB) is the ‘motor of cellular plasticity’ that shepherds epithelial-hybrid-mesenchymal transitions [29], and might be in slight disagreement with ‘equal’ decision-making potential of (miR-200/ZEB) and (miR-34/SNAIL) loops [95]. However, a detailed discussion on how specific the predictions are to the modeling framework adopted here vis-à-vis other modeling frameworks [17, 95] is outside the scope of this article, and is being dealt in sufficient detail, including experimental data, elsewhere (Jia *et al.* in preparation). Besides, a recent integrative study indicates that OVOL can mediate two intermediate states between E and M phenotypes - a hybrid E/M, and a ‘naïve’ one [17]. Conceptually, this observation is congruent with our proposition that OVOL expands the range of parameters (i.e. physiological conditions) for the existence of a hybrid E/M phenotype [16]. Moreover, the stable existence and functional significance of the proposed ‘naïve’ state [17] remains to be shown experimentally. Further accumulation of experimental evidence for intermediate state(s) is also expected to better integrate ‘bottom-up’ and ‘top-down’ modeling approaches that have been adopted to characterize the signature of hybrid E/M phenotype - the former considers a few ‘core’ components identified experimentally [97, 98] and focuses on elucidating their specific functions and the emergent outcome of their quantitatively-characterized interactions [17, 29, 95, 96], while the latter considers a much larger network and attempts to lay out all different possible steady states of the network [63, 99].

Overall, using a network-biology approach, we present three ‘phenotypic stability factors’ (PSFs) - OVOL, GRHL2, and miR-145 - that can maintain a hybrid E/M phenotype and have been predicted to increase the likelihood of hybrid E/M phenotype in gaining stemness. This proposed dual role of PSFs can be crucial during cancer metastasis as they can both enable collective migration of tumor cell clusters and confer these clusters with high tumor-initiating properties. The proposed contribution of PSFs to metastatic load is supported by lower metastasis-free survival, relapse-free survival, and overall survival time for patient samples overexpressing one or more of GRHL2, OVOL2, and CDH3. Targeting these PSFs therapeutically can help break the migrating CTC clusters that act as the primary ‘bad actors’ of metastasis because of the multiple advantages of cluster migration - resistance to anoikis, more tumor-initiating potential, ease of intravasation and extravasation, and finally the ‘priming’ for subsequent metastatic dissemination [5]. These results suggest a rethinking in the diagnostic strategy. Recent attempts have largely focused on isolating single CTCs [7], however, isolating and characterizing CTC clusters [100] might be the most effective and much-needed diagnostic approach [5].

Notably, the identification of PSFs presented here is by no means comprehensive. These examples studied here allow us to define a set of network motifs that will allow us to search for other PSFs. Specifically, we propose three particular network topologies that can be used to mine for other similar ‘phenotypic stability factors’ (PSFs) - (a) a double negative feedback loop with ZEB, (b) inhibition on both miR-200 and ZEB, and (c) a double negative feedback loop with ZEB as well as inhibiting miR-200. In all these three cases, the PSF can self-regulate positively or negatively. With a surging interest in mapping and modeling the signaling pathways regulating metastasis [45, 63, 101–104], the theoretical approach presented here can serve as a template to elucidate the effect of many intracellular and extracellular signals in regulating EMT dynamics and governing the relative stability of the E, M and E/M phenotypes.

## MATERIALS AND METHODS

### Cell line and siRNA transfection

The H1975 cell line was authenticated and free from mycoplasma, was grown in RPMI 1640 with 10% FBS and 1% penicillin/streptomycin. siRNA against GRHL2, OVOL2 and scrambled control siRNA were purchased from Sigma (Hs01_00105962, Hs01_00105964, Hs02_00357526, Hs01_00357580). The siRNA transfection was performed with Lipofectamine 2000 (catalog# 11668-019; Invitrogen), according to the manufacturer's instructions. An siRNA concentration of 50 nmol was used for a 35 mm dish of H1975 cells.

### Mathematical modeling of EMT signaling network and coupled EMT-stemness network

We mathematically modelled the coupling of the core EMT network (miR-200/ZEB) with GRHL2 and miR-145 (SI sections 1, 2) by generalizing and extending our previous theoretical framework [29]. We also coupled the core EMT and stemness modules (miR-200/ZEB and LIN28/let-7 respectively) along with GRHL2 (SI sections 1, 2) by extending our previous theoretical framework for EMT-stemness coupling [18, 45]. The parameters involved (SI Tables 1-3) in the model have been obtained from the literature or estimated from analyzing experimental data. The sensitivity analysis of the model (Figures S1–S2) indicates its robustness to parameter change. The number of molecules of miRNAs, mRNAs, and proteins has been estimated based on their typically observed concentrations in eukaryotic cells. For instance, the concentration of a protein is 10nM-1μM [105] which when multiplied with typical volume of a mammalian cell (100-1000 um^3^), amounts to around 6 million molecules, commensurate with the range depicted in the simulations in this paper. Similarly, the ratio of protein/mRNA of a particular gene can be ∼3000 [106], therefore the number of mRNA molecules of a gene can be estimated to be of the order of a thousand. In addition, the number of microRNA molecules in a cell is approximately10000 [107].

### RT-PCR analysis and immunofluorescence

Complementary DNA samples were prepared using a High-Capacity cDNA Reverse Transcription Kit (Life Technologies). A TaqMan PCR assay was performed with a 7500 Fast Real-Time PCR System using TaqMan PCR master mix, commercially available primers, FAM™-labeled probes for GRHL2, OVOL2, CDH1, Vimentin and VIC™-labeled probes for 18S, according to the manufacturer's instructions (Life Technologies). Each sample was run in triplicate. Ct values for each gene were calculated and normalized to Ct values for 18S (ΔCt). The ΔΔCt values were then calculated by normalization to the ΔCt value for control.

For immunofluorescence, cells were fixed in 4% paraformaldehyde, permeabilized in 0.2% Triton X-100, then stained with anti-CDH1 (1:100; Abcam) and anti-vimentin (1:100; Cell Signaling Technology). The primary antibodies were then detected with Alexa conjugated secondary antibodies (Life technologies). Nuclei were visualized by co-staining with DAPI.

### Wound-healing assay

A scratch or wound-healing assay was performed to determine cell migration using confluent cultures (80%−90% confluence). Briefly, cells (1 × 10^5^ cells/ml) were seeded in 6-well tissue culture plate and grown to confluence. Cells were starved for 24 hours using 0.2% serum in growth media and treated with mitomycin to minimize cell proliferation. The next day, the confluent monolayer was scratched with a sterile p200 pipet tip and media replaced with complete growth media. Images were acquired at 0 and 12 hours; the assay was performed at least twice per cell line. The quantification of area covered was done by ImageJ software.

### Kaplan-Meier plot analysis

Kaplan-Meier plots were generated using the online tool ProgGene [108]. The patients were classified into high or low based on the median level of expression for a given gene.

### Cell viability assay

Cells were seeded at 5000 cells/well in 96-well plates. After 24 hours, cells were transfected at a final concentration of 50 nM siRNA using Lipofectamine RNAiMAX (Life Technologies) according to the manufacturer's instructions. MTS assay (CellTiter 96 Aqueous One Solution Cell Proliferation Assay, Promega) was performed to assess the cell viability after 72 hours and 96 hours, according to the manufacturer's protocol.

## SUPPLEMENTARY MATERIALS FIGURES AND TABLES


