# Supplementary material for: Stability of the hybrid epithelial/mesenchymal phenotype
Source: Oncotarget. 2016 Mar 17;7(19):27067–84. doi: 10.18632/oncotarget.8166 (PMC5053633; doi:10.18632/oncotarget.8166)
Supplement: Supplementary file 1 [file oncotarget-07-27067-s001.pdf]

Stability of the hybrid epithelial/mesenchymal phenotype

Supplementary Information

1. Regulatory circuit details

(a) EMT regulatory network

The core EMT regulatory network includes two interconnected mutually inhibitory circuits – one between miR-34 and SNAIL, and the other between miR-200 and ZEB (1–3). Both these circuits have been shown to perform distinct and complementary roles in EMT decision-making, such that miR-34/SNAIL acts as a noise-buffering integrator, while miR-200/ZEB acts as a three-way decision-making switch allowing the co-existence of three phenotypes – epithelial (E - high miR-200, low ZEB), mesenchymal (M - low miR-200, high ZEB) and hybrid epithelial /mesenchymal (E/M - intermediate miR-200, intermediate ZEB)(4). Coupling miR-34/SNAIL to miR-200/ZEB does not change the qualitative behavior of EMT decision-making (4); hence we treat SNAIL as the external input signal to the miR-200/ZEB circuit.

The miR-200 family consists of two subgroups – one containing miR-141 and miR-200a, and the other with miR-200b, miR-200c, and miR-429. The ZEB transcription factor family includes two isoforms – ZEB1 and ZEB2. The 3' UTR region of ZEB1 has a total of eight conserved binding sites for miR-200 - three for the first subgroup and five for the second subgroup. 3' UTR region of ZEB2 has nine conserved binding sites for miR-200 (three for the first subgroup and six for the second subgroup) (5). Experiments suggest that the expression of miR-200c alone can restore E-cadherin and induce MET (6). Therefore, here, in our miR-200/ZEB module, we considered six binding sites (number of binding sites of the second subgroup on ZEB2) on the 3' UTR region of ZEB for the binding of miR-200. Also, the members of miR-200 family are located on two different chromosomes – miR-200c and miR-141 on chromosome 12 (with three conserved ZEB-binding sites); and miR-200b, miR-200a, and miR-141 on chromosome 1 (with two conserved ZEB-binding sites). We consider three binding sites for ZEB in the promoter region of miR-200. ZEB can also activate its own transcription both via stabilizing SMAD complexes (7) and by activating CD44s (8); hence we assume two binding sites for ZEB self-activation. SNAIL, a well-known EMT inducer, can transcriptionally inhibit miR-200 and activate ZEB (2,9). Both of these regulations have been assumed to happen through two binding sites (10). Also, miR-200c can be activated by EMT-inhibiting transcription factor p53 via two binding sites in its promoter region (11).

(b) Stemness regulatory network and its coupling with miR-200/ZEB

Stemness is governed by a mutually inhibitory loop between the families of RNA-binding factor LIN28 and microRNA let-7. LIN28A and LIN28B, the two members of LIN28 family, repress the biogenesis of

let-7 by blocking its processing to mature miRNAs. LIN28B inhibits the processing of primary let-7 transcripts by the microprocessor Drosha, and LIN28A recruits a TUTase (Zcchc11/ TUT4) to block the processing of let-7 precursors by Dicer in the cytoplasm (12,13). On the other hand, microRNA family let-7 can inhibit the translation process of LIN28 (12). Further, let-7 can promote its own processing and LIN28 can promote its own translation by binding to seven consensus sites on its own mRNA (14,15).

LIN28/let-7 circuit also behaves as a three-way switch – with the three states being U (Up – high LIN28, low let-7), D (Down – low LIN28, high let-7) and D/U (Down/Up – medium LIN28, medium let-7) (16). LIN28 promotes the translation of pluripotency factor OCT4 (17). Both very high and very low levels of OCT4 lead to differentiation, but its intermediate levels are strongly associated with gaining stemness (18–21). Therefore, cells with intermediate levels of LIN28 (or D/U state) are most likely to lie in the 'stemness window' or correspond with the maximum likelihood of gaining stemness. Finally, the two core networks – (miR-200/ZEB) and (LIN28/let-7) are coupled via two links: miR-200 inhibits LIN28 by binding to its mRNA, and let-7 inhibits HMGA2 that activates ZEB (22–26).

(c) Coupling of GRHL2 with (miR-200/ZEB)

GRHL2, a key regulator of morphogenesis (27), forms a mutually inhibitory loop with ZEB. Promoter region of GRHL2 has 3 binding sites for ZEB1, and that of ZEB1 has 1 binding site for GRHL2 (28,29).

(d) Coupling of (miR-145/OCT4) with (miR-200/ZEB)

miR-145 and OCT4 form a mutually inhibitory feedback loop, with one binding site of OCT4 on miR-145 promoter region, and one binding site of miR-145 on the 3' UTR of OCT4 mRNA (30). OCT4 can transcriptionally activate miR-200c via two binding sites (31), and miR-145 inhibits ZEB via one binding site in its 3' UTR, as predicted by TargetScan (32). The siRNA-mediated depletion of ZEB increases miR-145 two-to-three fold (33), so we assume two binding sites for the inhibition of miR-145 by ZEB.

2. Mathematical model formulation

(a) Theoretical framework for microRNA-based circuits (MBCs)

As described in our previous work (4), the terms representing miRNA-mediated translational effects capture both the degradation of miRNA - mRNA complex and the inhibition on translation of mRNA. A microRNA is usually 22nt long, and the seed sequence it recognizes on mRNA is only 7~8 nt long; here we assume the bindings of different microRNAs to be independent of one another. The binding rate between microRNA and mRNA is r_+ and the unbinding rate is r_-, and these binding/unbinding processes

between microRNA and mRNA are much faster as compared to production and degradation of proteins, therefore miRNA and mRNA are always in equilibrium, i.e. $r_+ \mu [m_i] = r_- [m_{i+1}]$, where μ represents the concentration of microRNA, $[m_i]$ represents the mRNA bound with i microRNAs and $[m_{i+1}]$ represents the mRNA bound with (i+1) microRNAs. Therefore, $[m_i] = \left(\mu / \mu_0\right)^i [m_0]$, where $\mu_0 = r_- / r_+$. All terms $[m_i]$ should satisfy $\sum_{i=0}^{n} C_n^i [m_i] = m$, where C_n^i is the number of combinations for *i* terms in *n* positions, which is defined as $C_n^i = n!/(i!(n-i)!)$ and m is total concentration of the mRNA.

Since there are C_n^i different possibilities for *i* number of microRNA (miR) molecules binding to mRNA with *n* binding sites for that particular microRNA (miR), therefore $[m_i] = m M_n^i(\mu)$, where $M_n^i(\mu) = \left(\mu / \mu_0\right)^i / \left(1 + \mu / \mu_0\right)^n$. Thus, the translation rate is given by $mL(\mu) = \sum_{i=0}^{n} l_i C_n^i [m_i] = m \sum_{i=0}^{n} l_i C_n^i M_n^i(\mu)$, where l_i is the translation rate of an mRNA when bound to *i* microRNAs. The term describing the degradation of the mRNA due to binding with the microRNA is $mY_m(\mu) = \sum_{i=0}^{n} \gamma_{mi} C_n^i [m_i] = m \sum_{i=0}^{n} \gamma_{mi} C_n^i M_n^i(\mu)$, where γ_{mi} is the individual degradation rate of mRNA bound to *i* molecules of miRNA. Similarly, the term denoting the degradation rate of microRNA due to binding to many mRNAs is $mY_\mu(\mu) = \sum_{i=0}^{n} i \gamma_{\mu i} C_n^i [m_i] = m \sum_{i=0}^{n} i \gamma_{\mu i} C_n^i M_n^i(\mu)$, where $\gamma_{\mu i}$ is the individual degradation rate for a microRNA molecule..

When the number of binding sites on the mRNA for miRNA is relatively small (~2), the miRNA-mediated regulation can be approximated as a Hill function (4).

Transcriptional regulation is denoted by shifted Hill functions $H^S(X,\lambda)$ defined as $H^S(X,\lambda) = H^-(X) + \lambda H^+(X)$, a weighted sum of positive and negative Hill functions. Parameter λ in the shifted Hill functions is the weight factor that represents fold-change in production rate from its basal level, due to the binding of regulatory factor. For activation, $\lambda > 1$ and shifted Hill function is represented by H^{S+}; for repression, $0 < \lambda < 1$ and shifted Hill function is represented by H^{S-}; and for no change, $\lambda = 1$. $\lambda_{X,Y}$ denotes the effect of X on Y(4).

(b) miR-200/ZEB/GRHL2 circuit

The miR-200/ZEB/GRHL2 circuit has five species - microRNA miR-200 (μ_{200}), ZEB mRNA (m_Z), ZEB protein (Z), GRHL2 mRNA (m_G), and GRHL2 protein (G). All of them have an innate production and degradation rate. The effects of miR-200 on ZEB are captured by both the degradation of mRNA by miRNAs (depicted by $Y_m(\mu)$) and the inhibition of translation by miRNAs (depicted by $L(\mu)$). Also, the miRNAs that bind to mRNAs can be degraded after forming a complex with mRNAs (depicted by $Y_\mu(\mu)$). The detailed derivation of these functions is presented in SI section 2 (a) and our earlier work on devising a theoretical framework for microRNA-based circuits(4); All parameters are given in Tables S1-3.

The dynamics of miR-200 (μ_{200}) can be described by the following equation:

$$\frac{d\mu_{200}}{dt} = g_{\mu_{200}} H^{S-}\left(Z,\lambda_{Z,\mu_{200}}\right) H^{S-}\left(S,\lambda_{S,\mu_{200}}\right) - m_Z Y_\mu\left(\mu_{200}\right) - k_{\mu_{200}}\mu_{200} \cdots\cdots(1)$$

where $g_{\mu_{200}}$ and $k_{\mu_{200}}$ are the innate production and degradation rates of miR-200 respectively. $H^{S-}\left(Z,\lambda_{Z,\mu_{200}}\right)$ represents the transcriptional inhibition of miR-200 by ZEB and $H^{S-}\left(S,\lambda_{S,\mu_{200}}\right)$ represents transcriptional inhibition of miR-200 by SNAIL. $Y_m\left(\mu_{200}\right)$ represents the degradation rate of miR-200 due to forming a complex with ZEB mRNAs.

When considering the effect of p53 on its downstream target miR-200, an additional shifted Hill function $H^{S+}\left(P,\lambda_{P,\mu_{200}}\right)$ is multiplied with the production rate of miR-200, and the updated equation is:

$$\frac{d\mu_{200}}{dt} = g_{\mu_{200}} H^{S-}\left(Z,\lambda_{Z,\mu_{200}}\right) H^{S-}\left(S,\lambda_{S,\mu_{200}}\right) H^{S+}\left(P,\lambda_{P,\mu_{200}}\right) - m_Z Y_\mu\left(\mu_{200}\right) - k_{\mu_{200}}\mu_{200} \cdots\cdots(1\_1)$$

The dynamics of ZEB mRNA (m_Z) and ZEB protein (Z) are described by the following equations:

$$\frac{dm_Z}{dt} = g_{m_Z} H^{S-}\left(G,\lambda_{G,m_z}\right) H^{S+}\left(Z,\lambda_{Z,m_Z}\right) H^{S+}\left(S,\lambda_{S,m_Z}\right) - m_Z Y_m\left(\mu_{200}\right) - k_{m_Z} m_Z \ldots\ldots\ldots\ldots\ldots\ldots(2)$$

$$\frac{dZ}{dt} = g_Z m_Z L\left(\mu_{200}\right) - k_Z Z \cdots\cdots(3)$$

where g_{m_Z} and g_Z are the innate production rates of ZEB mRNA and ZEB protein respectively, and k_{m_Z} and k_Z are their respective innate degradation rates. $H^{S+}\left(Z,\lambda_{Z,m_Z}\right)$ denotes transcriptional self-

activation of ZEB, and $H^{S+}\left(S,\lambda_{S,m_Z}\right)$ denotes transcriptional activation of ZEB by SNAIL. $Y_m\left(\mu_{200}\right)$ represents the degradation of ZEB mRNA due to forming mRNA-miRNA complexes with miR-200, $L\left(\mu_{200}\right)$ denotes the translational inhibition of ZEB by miR-200, and $H^{S-}\left(G,\lambda_{G,m_z}\right)$ represents the inhibition from GRHL2.

Dynamics of GRHL2 mRNA (m_G) and GRHL2 protein (G) are described by these equations:

$$\frac{dm_G}{dt}=g_{m_G}H^{S-}\left(Z,\lambda_{Z,m_G}\right)-k_{m_G}m_G.........(4)$$
$$\frac{dG}{dt}=g_Gm_G-k_GG...............................(5)$$

Where g_{m_G} and g_G are innate production rates of GRHL2 mRNA and protein respectively, and k_{m_G} and k_G are their respective degradation rates. $H^{S-}\left(Z,\lambda_{Z,m_G}\right)$ represents the transcriptional inhibition of GRHL2 by ZEB.

When considering an external activation (SA) or inhibition signal (SI) on GRHL2, shifted Hill functions $H^{S+}\left(SA,\lambda_{SA,m_G}\right)$ and $H^{S-}\left(SI,\lambda_{SI,m_G}\right)$ are respectively multiplied to the production term of GRHL2 mRNA:

$$\frac{dm_G}{dt}=g_{m_G}H^{S-}\left(Z,\lambda_{Z,m_G}\right)H^{S+}\left(SA,\lambda_{SA,m_G}\right)-k_{m_G}m_G.........(4)$$
$$or$$
$$\frac{dm_G}{dt}=g_{m_G}H^{S-}\left(Z,\lambda_{Z,m_G}\right)H^{S-}\left(SI,\lambda_{SI,m_G}\right)-k_{m_G}m_G.........(4)$$

(c) miR-200/ZEB/miR-145/OCT4 circuit

The miR-200/ZEB/miR-145/OCT4 circuit has six species - microRNA miR-200 (μ_{200}), ZEB mRNA (m_Z), ZEB protein (Z), miR-145 (μ_{145}), OCT4 mRNA (m_{O_C}), and OCT4 protein (O_C). All of them have an innate production and degradation rate. The effects of miR-200 on ZEB are captured similarly to as in the miR-200/ZEB/GRHL2 circuit; but the effect of miR-145 on OCT4 and ZEB are represented by

shifted Hill functions, because the silencing effect of microRNAs on their targets with a relatively smaller number of binding sites (~2) can be approximated as a Hill function (4).

The dynamics of miR-200 (μ_{200}) can be described by the following equation:

$$\frac{d\mu_{200}}{dt} = g_{\mu_{200}} H^{S-}\left(Z, \lambda_{Z,\mu_{200}}\right) H^{S-}\left(S, \lambda_{S,\mu_{200}}\right) H^{S+}\left(O_c, \lambda_{O_c,\mu_{200}}\right) - m_Z Y_\mu\left(\mu_{200}\right) - k_{\mu_{200}} \mu_{200} \cdots\cdots (6)$$

where $g_{\mu_{200}}$ and $k_{\mu_{200}}$ are the innate production and degradation rates of miR-200 respectively. $H^{S-}\left(Z, \lambda_{Z,\mu_{200}}\right)$ represents the transcriptional inhibition of miR-200 by ZEB, $H^{S-}\left(S, \lambda_{S,\mu_{200}}\right)$ represents the transcriptional inhibition of miR-200 by SNAIL, and $H^{S+}\left(O_c, \lambda_{O_c,\mu_{200}}\right)$ denotes the transcriptional activation of miR-200 by OCT4. $Y_m\left(\mu_{200}\right)$ represents the degradation rate of miR-200 due to forming a complex with ZEB mRNAs.

The dynamics of ZEB mRNA (m_Z) and ZEB protein (Z) are described by the following equations:

$$\frac{dm_Z}{dt} = g_{m_Z} H^{S-}\left(\mu_{145}, \lambda_{\mu_{145},m_z}\right) H^{S+}\left(Z, \lambda_{Z,m_Z}\right) H^{S+}\left(S, \lambda_{S,m_Z}\right) - m_Z Y_m\left(\mu_{200}\right) - k_{m_Z} m_Z \ldots\ldots\ldots\ldots\ldots\ldots (7)$$

$$\frac{dZ}{dt} = g_Z m_Z L\left(\mu_{200}\right) - k_Z Z \cdots\cdots (8)$$

where g_{m_Z} and g_Z are the innate production rates of ZEB mRNA and ZEB protein respectively, and k_{m_Z} and k_Z are their respective innate degradation rates. $H^{S+}\left(Z, \lambda_{Z,m_Z}\right)$ denotes the transcriptional self-activation of ZEB, $H^{S+}\left(S, \lambda_{S,m_Z}\right)$ denotes the activation of ZEB by SNAIL, and $H^{S-}\left(\mu_{145}, \lambda_{\mu_{145},m_z}\right)$ denotes the inhibition of ZEB by miR-145. $Y_m\left(\mu_{200}\right)$ represents the degradation of ZEB mRNA due to forming mRNA-miRNA complexes with miR-200, $L\left(\mu_{200}\right)$ denotes the inhibition of ZEB by miR-200.

The dynamics of miR-145 (μ_{145}), OCT4 mRNA (m_{O_C}), and OCT4 protein (O_C) are given by:

$$\frac{dm_{O_c}}{dt} = g_{m_{O_c}} H^{S-}\left(\mu_{145}, \lambda_{\mu_{145}, m_{O_c}}\right) - k_{m_{O_c}} m_{O_c} \text{.........}(9)$$

$$\frac{dO_c}{dt} = g_{O_c} m_{O_c} - k_{O_c} O_c \text{.....................................}(10)$$

$$\frac{d\mu_{145}}{dt} = g_{\mu_{145}} H^{S-}\left(Z, \lambda_{Z,\mu_{145}}\right) H^{S-}\left(O_c, \lambda_{\mu_{145}, m_{O_c}}\right) - k_{\mu_{145}} \mu_{145} \text{.....}(11)$$

where $g_{m_{O_c}}$, g_{O_c} and $g_{\mu_{145}}$ represent the innate production rates for OCT4 mRNA, OCT4 protein and miR-145 respectively; and $k_{m_{O_c}}$, k_{O_c} and $k_{\mu_{145}}$ represent their respective innate degradation rates. $H^{S-}\left(\mu_{145}, \lambda_{\mu_{145}, m_{O_c}}\right)$ represents the inhibition of OCT4 by miR-145; $H^{S-}\left(Z, \lambda_{Z,\mu_{145}}\right)$ and $H^{S-}\left(O_c, \lambda_{\mu_{145}, m_{O_c}}\right)$ denote the inhibition of miR-145 by ZEB and OCT4 respectively.

miR-145 can be activated by p53 via two binding sites in its promoter region (34). When considering the effect of p53 on its target miR-145, an additional shifted Hill function is multiplied with the production rate of miR-145, and the updated equation is:

$$\frac{d\mu_{145}}{dt} = g_{\mu_{145}} H^{S-}\left(Z, \lambda_{Z,\mu_{145}}\right) H^{S-}\left(O_c, \lambda_{\mu_{145}, m_{O_c}}\right) H^{S+}\left(P, \lambda_{P,\mu_{145}}\right) - k_{\mu_{145}} \mu_{145} \text{.....}(11\_1)$$

where $H^{S+}\left(P, \lambda_{P,\mu_{145}}\right)$ denotes the activation of miR-145 by p53.

To evaluate the effect of p53 on miR-200, an additional shifted Hill function $H^{S+}\left(P, \lambda_{P,\mu_{200}}\right)$ denoting the effect of p53 on miR-200 is multiplied with the production rate of miR-200. The updated equation is:

$$\frac{d\mu_{200}}{dt} = g_{\mu_{200}} H^{S-}\left(Z, \lambda_{Z,\mu_{200}}\right) H^{S-}\left(S, \lambda_{S,\mu_{200}}\right) H^{S+}\left(O_c, \lambda_{O_c,\mu_{200}}\right) H^{S+}\left(P, \lambda_{P,\mu_{200}}\right) - m_Z Y_\mu\left(\mu_{200}\right) - k_{\mu_{200}} \mu_{200} \cdots\cdots(6\_1)$$

(d) miR-200/ZEB/LIN28/let-7/GRHL2 circuit

The miR-200/ZEB/LIN28/let-7/GRHL2 circuit has multiple modes of regulation – transcriptional, translational, miRNA-mediated regulation, and regulation of miRNA processing. For all modes except miRNA-mediated regulation with a large number of binding sites of miRNA on its target mRNA, shifted Hill functions $H^S(X, \lambda)$ (4) are used to denote the regulation; thus, when let-7 inhibits LIN28 and ZEB,

and miR-200 inhibits LIN28, shifted Hill functions are used to represent the effects of microRNAs. Also, inhibition of let-7 by LIN28 has been shown experimentally to behave as an inhibitory Hill function (35).

Specifically in the two links that couple EMT and stemness core circuits – miR-200 inhibiting LIN28 and let-7 inhibiting ZEB – the strength of the inhibition is defined as $\alpha = 1 - \lambda$, $0 < \alpha < 1$, therefore the larger value of α represents stronger inhibition. A shifted Hill function formula for these two inhibitions can thus be denoted by $H^{S-}(B,\alpha) = H^{-}(B) + \lambda H^{+}(B) = 1 - \alpha H^{+}(B)$, where $0 < \alpha < 1$.

This circuit contains eight components - microRNA miR-200 (μ_{200}), ZEB mRNA (m_Z), ZEB protein (Z), microRNA let-7 (μ_7), LIN28 mRNA (m_l), LIN28 protein (L), GRHL2 mRNA (m_G), and GRHL2 protein (G). The dynamics of miR-200 (μ_{200}), GRHL2 mRNA (m_G) and GRHL2 protein (G) are described by equations (1), (4), and (5) respectively.

The dynamics of ZEB mRNA (m_Z) and ZEB protein (Z) are described by the following equations:

$$\frac{dm_Z}{dt} = g_{m_Z} H^{S+}\left(Z, \lambda_{Z,m_Z}\right) H^{S+}\left(S, \lambda_{S,m_Z}\right) H^{S-}\left(G, \lambda_{G,m_Z}\right) - m_Z Y_m\left(\mu_{200}\right) - k_{m_Z} m_Z \cdots\cdots (12)$$

$$\frac{dZ}{dt} = g_Z m_Z L\left(\mu_{200}\right) H^{S-}\left(u_l, \alpha_{u_l,m_Z}\right) - k_Z Z \cdots\cdots (13)$$

where g_{m_Z} and g_Z are the innate production rates of ZEB mRNA and ZEB protein respectively, and k_{m_Z} and k_Z are their respective innate degradation rates. $H^{S+}\left(Z, \lambda_{Z,m_Z}\right)$ denotes transcriptional self-activation of ZEB, $H^{S+}\left(S, \lambda_{S,m_Z}\right)$ denotes transcriptional activation of ZEB by SNAIL, $H^{S-}\left(G, \lambda_{G,m_Z}\right)$ denotes transcriptional inhibition of ZEB by GRHL2. $Y_m\left(\mu_{200}\right)$ represents the degradation of ZEB mRNA due to forming mRNA-miRNA complexes with miR-200, $L\left(\mu_{200}\right)$ denotes the translational inhibition of ZEB by miR-200, and $H^{S-}\left(u_l, \alpha_{u_l,m_Z}\right)$ represents the inhibition from let-7.

The dynamics of let-7 (μ_7) is described by the following equation:

$$\frac{d\mu_7}{dt} = g_{\mu_7} H^{S+}\left(\mu_l, \lambda_{\mu_7,\mu_7}\right) H^{S-}\left(L, \lambda_{L,\mu_l}\right) H^{S+}(N, \lambda_{N,m_l}) - k_{\mu_7}\mu_7 \ldots\ldots\ldots\ldots (14)$$

where g_{μ_7} and k_{μ_7} are the innate production and degradation rates of let-7 respectively, $H^{S+}\left(\mu_7, \lambda_{\mu_7,\mu_7}\right)$ represents the self-activation of let-7, $H^{S-}\left(L, \lambda_{L,\mu_7}\right)$ represents inhibition by protein LIN28 and $H^{S+}(N, \lambda_{N,\mu_7})$ denotes activation by external signal (protein NF-kB (represented by N)).

Dynamics of LIN28 mRNA (m_l) and protein (L) are described by the following equations:

$$\frac{dm_l}{dt} = g_{m_O} - k_{m_l} m_l \text{..}(15)$$

$$\frac{dL}{dt} = g_L m_l H^{S-}\left(\mu_{200}, \alpha_{u_{200},L}\right) H^{S+}\left(L, \lambda_{L,L}\right) H^{S+}(N, \lambda_{N,L}) H^{S-}(\mu_7, \lambda_{\mu_7,L}) - k_L L\text{.........}(16)$$

where g_{m_l} and g_L are the innate production rates of LIN28 mRNA and protein respectively, and k_{m_l} and k_L are their respective degradation rates. $H^{S+}\left(L, \lambda_{L,L}\right)$ denotes the translational self-activation of LIN28, $H^{S+}\left(N, \lambda_{N,L}\right)$ represents the transcriptional activation of LIN28 by external signal (NF-kB (denoted by N)), $H^{S-}\left(\mu_{200}, \alpha_{u_{200},L}\right)$ denotes the inhibition of LIN28 by miR-200, and $H^{S-}(\mu_7, \lambda_{\mu_7,L})$ denotes the inhibition of LIN28 by let-7.

(g) miR-200/ZEB/I and miR-200/ZEB/I/LIN28/let-7/OCT4 circuits

An incoherent external signal I can be coupled to miR-200/ZEB circuit by incorporating respective shifted Hill functions, as shown in equation (21) and (22).

$$\frac{dm_Z}{dt} = g_{m_Z} H^{S-}\left(I, \lambda_{I,m_z}\right) H^{S+}\left(Z, \lambda_{Z,m_Z}\right) H^{S+}\left(S, \lambda_{S,m_Z}\right) - m_Z Y_m\left(\mu_{200}\right) - k_{m_Z} m_Z \text{............}(21)$$

$$\frac{d\mu_{200}}{dt} = g_{\mu_{200}} H^{S-}\left(I, \lambda_{I,\mu_{200}}\right) H^{S-}\left(Z, \lambda_{Z,\mu_{200}}\right) H^{S-}\left(S, \lambda_{S,\mu_{200}}\right) - m_Z Y_\mu\left(\mu_{200}\right) - k_{\mu_{200}} \mu_{200} \cdots\cdots(22)$$

The miR-200/ZEB/I circuit can be coupled to LIN28/let-7 in the exact same manner as described for the previous case – miR-200/ZEB/GRHL2.

3. Parameter estimation

The estimation of the levels for miRNAs, mRNAs, and proteins are made according to their canonical concentrations in eukaryotic cells. Typically, the volume of a mammalian cell is 100-10000 um^3 and the concentration for a single protein is 10nM-1μM (36). 1μM protein concentration amounts to around 6 million molecules ($6.02 \times 10^{23} \times 10^{-6} \times \left(10000 \times \left(10^{-5}\right)^3\right)$) for a typical eukaryotic cell. The ratio of protein/mRNA for one gene is about 2800 (37), hence the number of mRNA molecules for one gene should be around 1000. Number of microRNA molecules in a cell are around 10000 molecules (38), hence μ_0 =10000 molecules. The translation rate for one gene is around 140 proteins per mRNA per hour (37), so we used 100 proteins per ZEB or OCT4 mRNA and 200 proteins per GRHL2 mRNA as their translation rates. Innate degradation rates of miRNAs, mRNAs, and proteins were selected based on their half-lives from experimental data. Typically, the half-life of mammalian proteins is about 10 hours (39); therefore we selected 0.1 $hour^{-1}$ as the innate degradation rate for proteins ZEB, GRHL2 and OCT4. The half-life of mRNA is a few hours (40), so we chose 0.5 $hour^{-1}$ as the innate degradation rate for ZEB mRNA and GRHL2 mRNA, and 0.1 $hour^{-1}$ as that for OCT4 mRNA. The innate degradation rate of miR-200 and miR-145 was selected as 0.05 $hour^{-1}$ and 0.01$hour^{-1}$ respectively, as generally miRNAs are more stable than mRNAs (41,42). For transcriptional regulation, the weight factors for shifted Hill functions vary from 5 to 10 for activation, and from 0.5 to 0.1 for repression.

**Species**	**Production rates (molecules/Hour)**		**Degradation rates ($Hour^{-1}$)**	
miR-200	$g_{\mu_{200}}$	2100	$k_{\mu_{200}}$	0.05
ZEB mRNA	g_{m_Z}	11	k_{m_Z}	0.5
ZEB protein	g_Z	100	k_Z	0.1
let-7	g_{μ_7}	200	k_{μ_7}	0.05
LIN28 mRNA	g_{m_L}	100	k_{m_L}	0.5
LIN28 protein	g_L	200	k_L	0.1
OCT4 mRNA	$g_{m_{O_c}}$	60	$k_{m_{O_c}}$	0.1
OCT4 protein	g_{O_c}	80	k_{O_c}	0.1
miR-145	$g_{\mu_{145}}$	1000	$k_{\mu_{145}}$	0.01
GRHL2 mRNA	g_{m_G}	22	k_{m_G}	0.5
GRHL2 protein	g_G	200	k_G	0.1

Table S1. The production and degradation rates of different species in the circuits

Description	Fold change	Value	# binding sites	Value	Threshold	Molecules
Inhibition of ZEB by GRHL2	λ_{G,m_Z}	0.1	n_{G,m_Z}	1	$G^0_{m_Z}$	25000
Inhibition of GRHL2 by ZEB	λ_{Z,m_G}	0.5	n_{Z,m_G}	3	$Z^0_{m_G}$	10000
Self-activation of ZEB	l_{Z,m_Z}	7.5	n_{Z,m_Z}	2	$Z^0_{m_z}$	25000
Activation of miR-200 by p53	$\lambda_{P,\mu_{200}}$	5	$n_{P,\mu_{200}}$	2	$P^0_{\mu_{200}}$	150000
Inhibition of miR-200 by ZEB	$l_{Z,m_{200}}$	0.1	$n_{Z,m_{200}}$	3	$Z^0_{m_{200}}$	220000
Activation of ZEB by SNAIL	l_{S,m_Z}	10	n_{S,m_Z}	2	$S^0_{m_z}$	180000
Inhibition of miR-200 by SNAIL	$l_{S,m_{200}}$	0.1	$n_{S,m_{200}}$	2	$S^0_{m_{200}}$	180000
Inhibition of OCT4 by miR-145	$\lambda_{\mu_{145},m_{O_c}}$	0.1	$n_{\mu_{145},m_{O_c}}$	1	$\mu^0_{145\,m_{O_c}}$	15000
Inhibition of miR-145 by OCT4	$\lambda_{O_c,\mu 145}$	0.5	$n_{O_c,\mu 145}$	1	$O^0_{c\mu_{145}}$	500000
Activation of miR-145 by p53	$\lambda_{P,\mu_{145}}$	5	$n_{P,\mu_{145}}$	2	$P^0_{\mu_{145}}$	150000
Activation of miR-200 by OCT4	$\lambda_{O_c,\mu 200}$	5	$n_{O_c,\mu 200}$	2	$O^0_{c\mu_{200}}$	500000
Inhibition of miR-145 by ZEB	$\lambda_{Z,\mu_{145}}$	0.1	$n_{Z,\mu_{145}}$	1	$Z^0_{\mu_{145}}$	100000
Inhibition of ZEB by miR-145	λ_{μ_{145},m_Z}	0.5	n_{μ_{145},m_Z}	1	$\mu^0_{145\,m_Z}$	1000
Self-activation of let-7	λ_{μ_7,μ_7}	11	n_{μ_7,μ_7}	3	$\mu^0_{7\,\mu_7}$	12000
Inhibition of LIN28 by let-7	$\lambda_{\mu_7,L}$	0.1	$n_{\mu_7,L}$	1	$\mu^0_{7_L}$	25000
Self-activation of LIN28	$\lambda_{L,L}$	3	$n_{L,L}$	7	L^0_L	300000
Inhibition of let-7 by LIN28	λ_{L,μ_7}	0.1	n_{L,μ_7}	2	$L^0_{\mu_7}$	500000
Activation of OCT4 by LIN28	Positive Hill function		n_{L,O_c}	2	$L^0_{O_c}$	430000
External signal (SA/SI) on GRHL2	λ_{SA,m_G} (λ_{SI,m_G})	2 (0.2)	$n_{SA,m_G} = n_{SI,m_G}$	2	$SA^0_{m_G} = SI^0_{m_G}$	100000
Activation of let-7 by NF-kB	λ_{N,μ_7}	2	n_{N,μ_7}	2	$N^0_{\mu_7}$	25000
Activation of LIN28 by NF-kB	$\lambda_{N,L}$	2	$n_{N,L}$	2	N^0_L	250000

Inhibition of ZEB by I	λ_{I,m_Z}	0.3	n_{I,m_Z}	1	$I^0_{m_z}$	4000
Inhibition of miR-200 by I	$\lambda_{I,\mu_{200}}$	0.6	$n_{I,\mu_{200}}$	1	$I^0_{\mu_{200}}$	4000

Table S2. The parameters used in different Hill functions for different circuits

**n (# of miRNA binding sites)**	0	1	2	3	4	5	6
l_i **(hour^{-1})**	1	0.6	0.3	0.1	0.05	0.05	0.05
g_{mi} **(hour^{-1})**	0	0.04	0.2	1	1	1	1
g_m**(hour^{-1})**	0	0.005	0.05	0.5	0.5	0.5	0.5

Table S3. The parameters used in Y(u), Y(m) and L functions.

The two coupling links between EMT and stemness circuits are modeled as shifted Hill functions. For the feed-forward coupling (miR-200 inhibiting LIN28), miR-200 has 2 binding sites on LIN28 and threshold levels of miR-200 required to inhibit LIN28 are 25000 molecules. For the feed-backward coupling (let-7 inhibiting ZEB), let-7 has 2 binding sites on ZEB and threshold levels of let-7 are 50000 molecules. For both these links, coupling strengths α1 and α2 lie between 0 and 1; and are specified wherever the coupled miR-200/ZEB/LIN28/let-7 circuit is simulated at a particular value of (α1, α2). For hypothetical self-activation of GRHL2 (Figure 8C), n_{G,m_G} =2 and $G^0_{m_G}$ =40000 molecules. Also, for the hypothetical inhibition of miR-200 by GRHL2 (Figure 8D), $n_{G,\mu_{200}}$ =1 and $G^0_{\mu_{200}}$ =80000 molecules.

4. Parameter sensitivity analysis

To test the sensitivity of our predictions based on the parameters listed above, we conduct parameter sensitivity analysis by varying each parameter at one time. All the parameters – production rates, degradation rates, thresholds and weight factors in the shifted Hill functions are varied by +/-10%. The number of binding sites for the different interactions have been kept fixed as most of them are directly determined from experimental data. Sensitivity of the model is measured by how changes in the parameters affect the range of SNAIL levels for which the hybrid E/M phenotype exists (either alone in or combination with other phenotypes). Our previous analysis shows that the model for (miR-200/ZEB) circuit is sensitive towards low values of miR-200, i.e. changes in the parameters which decrease the levels of miR-200 decrease the overall range of SNAIL levels for the existence of the E/M phenotype(43). Here, we perform a similar analysis for the miR-200/ZEB/GRHL2 and miR-200/ZEB/miR-145/OCT4 circuits where we plot the range of SNAIL levels for the existence of the hybrid E/M phenotype, for

increase or decrease of every parameter; then comparing it with the control case (no parameter increased/ decreased).

(a) Parameter sensitivity analysis for miR-200/ZEB/GRHL circuit

Coupling GRHL2 with miR-200/ZEB enlarges the range of SNAIL levels for the existence of a hybrid E/M region (Figure 3A, B). We observe that when parameters are increased or decreased by 10%, the absolute levels of SNAIL for which the hybrid E/M phenotype exists changes in most cases (Figure S1). However, here we focus on the change in the range of SNAIL levels for the existence of the hybrid E/M state, when different parameters are varied. Compared with the control case (no parameter is changed – case 0 in Figure S1A-C), the range of SNAIL levels for the existence of an E/M state decreases significantly when the production rate for ZEB mRNA is increased (case B1), the degradation rate for ZEB mRNA is decreased (case G2) (Figure S1A), threshold of ZEB levels for the shifted Hill function of inhibition of ZEB on miR-200 is decreased (case K2) (Figure S1B), and the strength of ZEB activation by SNAIL is increased (case T1), and the strength of ZEB self-activation is increased (case W1) (Figure S1C). All these cases represent cases where the effective ZEB levels that can inhibit miR-200 and drive EMT are increased, i.e. the propensity of the cell to undergo EMT is increased. Also, consistently, at increased ZEB levels, GRHL2 levels are decreased (ZEB inhibits GRHL2) and hence the effect of

GRHL2 in expanding the hybrid E/M range is decreased.

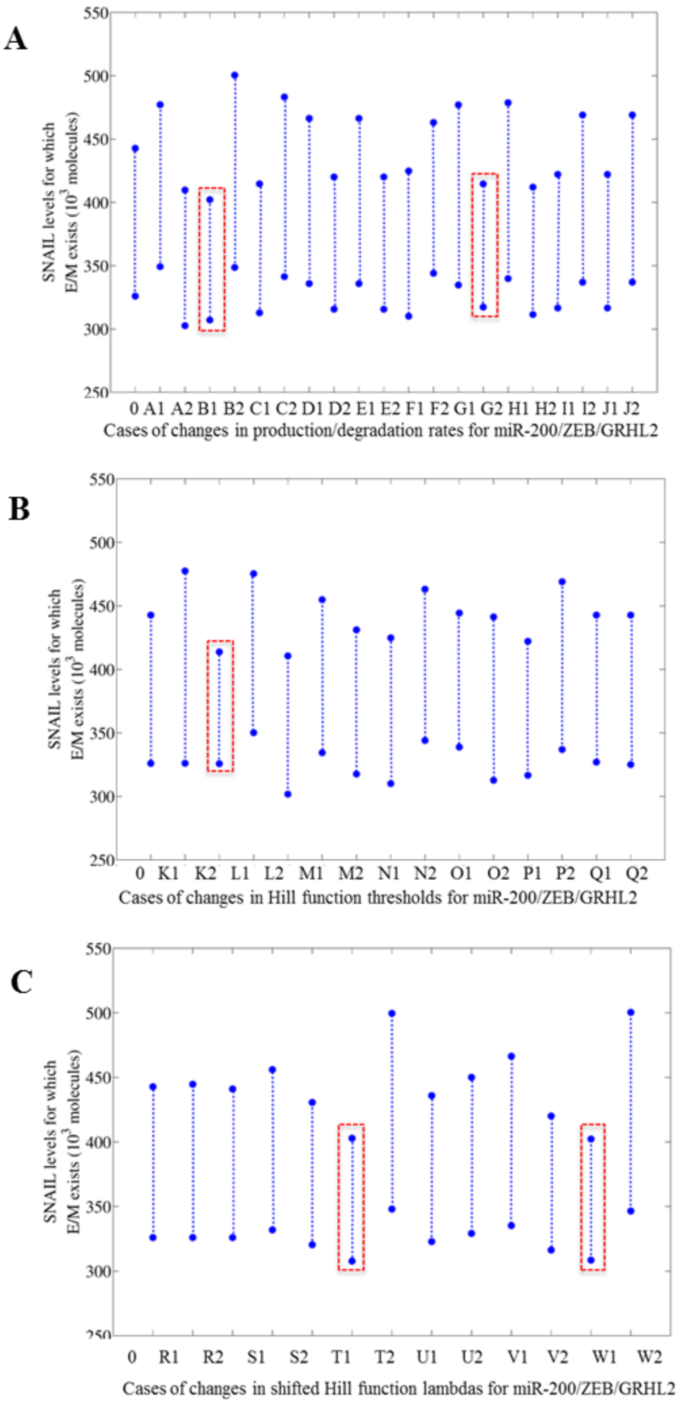

Figure S1 Parameter sensitivity analysis for the miR-200/ZEB/GRHL2 circuit (at p53=0) driven by the input signal SNAIL. Alphanumeric codes on x-axis represent the cases with different changed parameters by +/- 10%. Number 1 after the alphanumeric code represents the increase of the parameter by 10% and number 2 after the alphanumeric code represents the decrease of the parameter by 10%. Case 0 represents the result in this work, which is the control case. (**A) represents the cases with +/- 10% changes in the production and degradation rates.** A1 and A2 represent the increase and decrease in the

production rate of miR-200 (denoted by $g_{\mu_{200}}$) by 10% respectively. B1 and B2 represent the increase and decrease in the production rate of ZEB mRNA by 10% (denoted by g_{m_Z}) respectively. C1 and C2 represent the increase and decrease in the production rate of ZEB protein (denoted by g_Z) by 10% respectively. D1 and D2 represent respectively the increase and decrease in the production rate of GRHL2 (denoted by g_{m_G}) by 10%. E1 and E2 represent the increase and decrease in production rate of GRHL2 (denoted by g_G) by 10%. F1 and F2 represent the increase and decrease in the degradation rate of miR-200 (denoted by $k_{\mu_{200}}$) by 10% respectively. G1 and G2 represent the increase and decrease in the degradation rate of ZEB mRNA by 10% (denoted by k_{m_Z}) respectively. H1 and H2 represent the increase and decrease in degradation rate of ZEB protein (denoted by k_Z) by 10% respectively. I1 and I2 represent respectively the increase and decrease in degradation rate of GRHL2 (denoted by k_{m_G}) by 10%. J1 and J2 represent the increase and decrease in the degradation rate of GRHL2 (denoted by k_G) by 10%. **(B) represents the cases with changes in the thresholds in the shifted Hill functions.** K1 and K2 represent respectively the increase and decrease in the threshold levels for the inhibition of miR-200 by ZEB (denoted by $Z^0_{m_{200}}$) by 10%. L1 and L2 represent the 10% increase and decrease in the threshold levels of SNAIL inhibition on miR-200 (denoted by $S^0_{m_{200}}$) respectively. M1 and M2 represent the increase and decrease in the threshold levels of SNAIL activation on ZEB (denoted by $S^0_{m_Z}$) by 10%. N1 and N2 represent the increase and decrease in the threshold levels of miR-200 (denoted by m^0_{200}) by 10%. O1 and O2 represent the case for 10% increase and decrease in threshold levels of ZEB for its self-activation (denoted by $Z^0_{m_Z}$). P1 and P2 represent 10% increase and decrease in threshold levels of GRHL2 for inhibition of ZEB (denoted by $G^0_{m_Z}$) and Q1 and Q2 denote 10% increase and decrease in threshold levels of ZEB for inhibiting GRHL2 (denoted by $Z^0_{m_G}$). **(C) represents the cases with changes in the weight factors in the shifted Hill functions.** R1 and R2 denote the increase and decrease in the weight factor of ZEB inhibition on miR-200 (denoted by $\lambda_{Z,m_{200}}$) by 10%. S1 and S2 represent the respective increase and decrease in the weight factor of SNAIL inhibition on miR-200 (denoted by $\lambda_{S,m_{200}}$) by 10%. T1 and T2 denote respective increase and decrease in weight factor of SNAIL activation on ZEB (denoted by λ_{S,m_Z}). U1 and U2 denote the respective increase and decrease in weight factor of GRHL2 inhibition of ZEB (denoted by λ_{G,m_Z}) by 10%. T1 and T2 denote the respective increase and decrease in weight factor of inhibition of GRHl2 by ZEB (denoted by λ_{Z,m_G}) by 10%. U1 and U2 represent the respective increase and decrease in the weight factor of ZEB self-activation (denoted by λ_{Z,m_Z}) by 10%. Dotted boxes represent cases when the range of SNAIL levels for the existence of hybrid E/M phenotype is affected largely – either decreased or increased.

Conversely, the parameter changes that are likely to decrease the effective ZEB levels that can induce EMT should enlarge the range of SNAIL levels for which the hybrid E/M state exists. This is indeed observed for cases such as when the production rates of ZEB mRNA and ZEB protein are decreased

(cases B2, C2), the threshold of ZEB levels for the shifted Hill function of inhibition of ZEB on miR-200 is increased (case K1), and the strength of ZEB activation by SNAIL is decreased (case T2), and the strength of ZEB self-activation is decreased (case W2) (Figure S1). Thus, a change in several parameters of the miR-200/ZEB/GRHL2 circuit, especially those affecting the ZEB protein levels, can affect the range of SNAIL levels for which the hybrid E/M state exists. The change of parameters with respect to GRHL2 did not affect this range.

Therefore, for most parameter changes (either increase or decrease), the region of SNAIL levels for which the hybrid E/M exists does not change much, as long as the ZEB levels are not very high, thereby suggesting that our prediction regarding the 'phenotypic stability factor' role of GRHL2 is quite robust to parameter variation. Furthermore, the role of GRHL2 in increasing the association of the E/M phenotype with stemness is also likely to be robust to parameter changes, as long as let-7 levels are not too low, because similar to miR-200/ZEB, LIN28/let-7 is sensitive towards the low levels of let-7 (44). Low levels of let-7 would again imply higher levels of ZEB, and therefore low levels of GRHL2, thereby attenuating the role of GRHL2 in stabilizing the E/M phenotype as well as increasing its association with stemness. Next, we conduct a similar sensitivity analysis for the miR-200/ZEB/miR-145/OCT4 circuit.

(b) Parameter sensitivity analysis for miR-200/ZEB/miR-145/OCT4 circuit

Coupling miR-145/OCT4 with miR-200/ZEB enlarges the range of SNAIL levels for the existence of the hybrid E/M region (Figure 3A, C). We observe that for 10% increase or decrease in the parameters, the absolute levels of SNAIL for which the hybrid E/M phenotype exists changes mildly in most cases (Figure S2). However, focusing on the change in the range of SNAIL levels for the existence of hybrid E/M state, we find that compared with the control case (no parameter changed – case 0 in Figure S1A-C), the range of SNAIL for the existence of E/M state decreases relatively significantly when the production rate of ZEB mRNA is increased (case B1) or its degradation rate is decreased (case H2) (Figure S2A), threshold of ZEB levels for the shifted Hill function of inhibition of ZEB on miR-200 is decreased (case M2) (Figure S1B), and the strength of ZEB activation by SNAIL is increased (case Y1), strength of ZEB self-activation is increased (case AB1), and the strength of miR-145 inhibiting ZEB is weakened (case AC1) (Figure S1C). All these cases represent cases where the effective ZEB levels that can inhibit miR-200 and drive EMT are increased, i.e. the propensity of the cell to undergo EMT is increased. Also, consistently, at increased ZEB levels, miR-145 levels are decreased (ZEB inhibits miR-145) and hence the effect of miR-145 in expanding the hybrid E/M range is decreased.

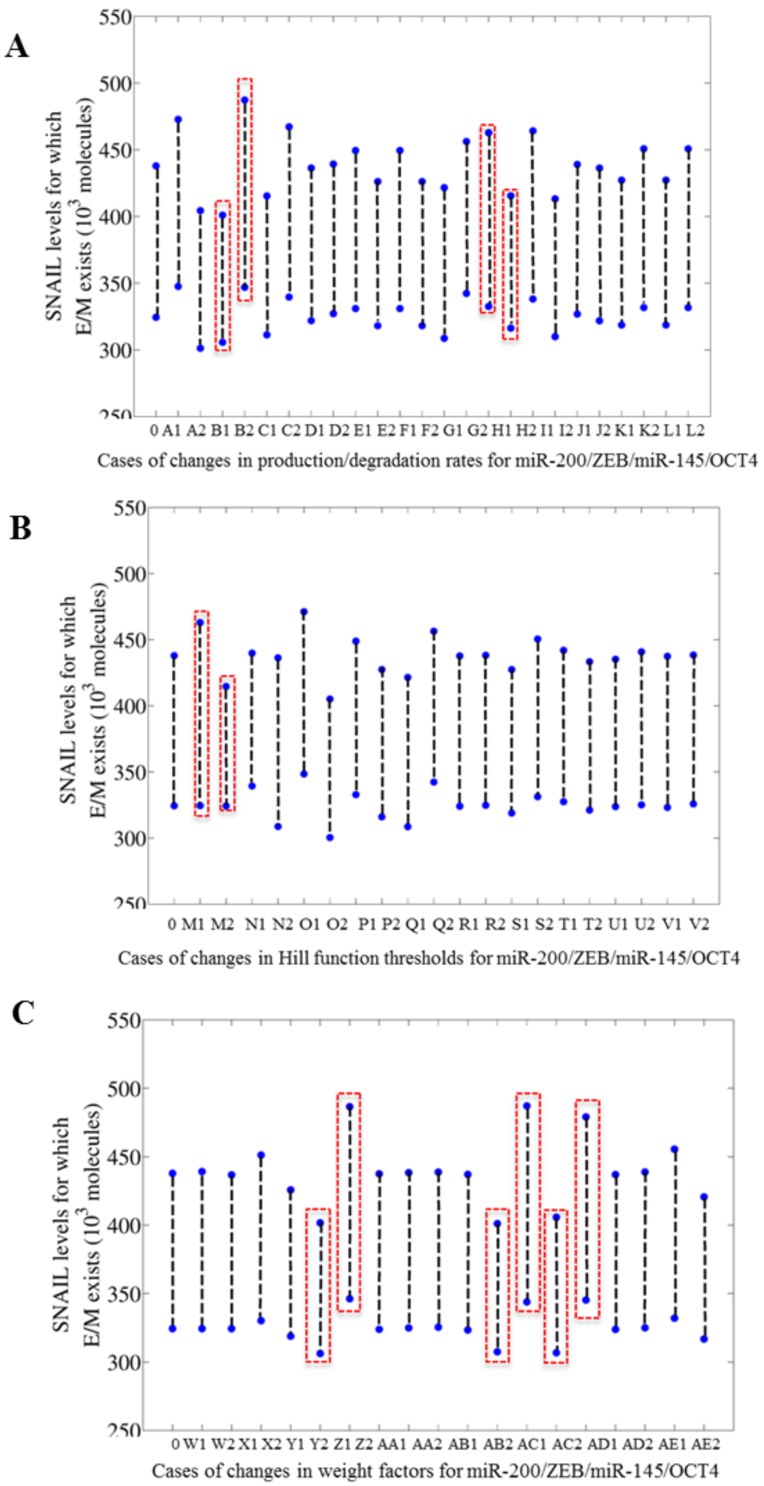

Figure S2 Parameter sensitivity analysis for the miR-200/ZEB/miR-145/OCT4 circuit (at p53=0) driven by the input signal SNAIL. Alphanumeric codes on x-axis represent the cases with different changed parameters by +/- 10%. Number 1 after the alphanumeric code represents the increase of the parameter by 10% and number 2 after the alphanumeric code represents the 10% decrease in parameter. Case 0 represents the result in this work, which is the control case. (**A) represents the cases with +/- 10%**

changes in the production and degradation rates. A1 and A2 represent the increase and decrease in the production rate of miR-200 (denoted by $g_{\mu_{200}}$) by 10% respectively. B1 and B2 represent the increase and decrease in production rate of ZEB mRNA by 10% (denoted by g_{m_Z}). C1 and C2 represent the increase and decrease in production rate of ZEB protein (denoted by g_Z) by 10%. D1 and D2 represent increase and decrease in the production rate of miR-145 (denoted by $g_{\mu_{145}}$) by 10%. E1 and E2 represent increase and decrease in production rate of OCT4 mRNA (denoted by $g_{m_{O_c}}$) by 10%. F1 and F2 represent increase and decrease in the degradation rate of OCT4 protein (denoted by g_{O_c}) by 10%. G1 and G2 represent increase and decrease in the degradation rate of miR-200 by 10% (denoted by $k_{\mu_{200}}$). H1 and H2 represent increase and decrease in degradation rate of ZEB mRNA by 10% (denoted by k_{m_Z}). I1 and I2 represent increase and decrease in degradation rate of ZEB protein (denoted by k_Z) by 10%. J1 and J2 represent respectively the increase and decrease in degradation rate of miR-145 (denoted by $k_{\mu_{145}}$) by 10%. K1 and K2 represent the increase and decrease in degradation rate of OCT4 mRNA (denoted by $k_{m_{O_c}}$) by 10%. L1 and L2 represent increase and decrease in degradation rate of OCT4 protein (denoted by k_{O_c}) by 10%. **(B) represents the cases with changes in the thresholds in the shifted Hill functions.** M1 and M2 represent increase and decrease in the threshold levels for the inhibition of miR-200 by ZEB (denoted by $Z^0_{m_{200}}$) by 10%. N1 and N2 represent the case for 10% increase and decrease in threshold levels of ZEB for self-activation (denoted by $Z^0_{m_Z}$). O1 and O2 represent the 10% increase and decrease in the threshold levels of SNAIL inhibition on miR-200 (denoted by $S^0_{m_{200}}$) respectively. P1 and P2 represent the increase and decrease in the threshold levels of SNAIL activation on ZEB (denoted by $S^0_{m_Z}$) by 10%. Q1 and Q2 represent the increase and decrease in the threshold levels of miR-200 (denoted by m^0_{200}) by 10%. R1 and R2 represent 10% increase and decrease in threshold levels of OCT4 for inhibiting miR-145 (denoted by $O^0_{c_{\mu_{145}}}$). S1 and S2 denote 10% increase and decrease in threshold levels of OCT4 for activating miR-200 (denoted by $O^0_{c_{\mu_{200}}}$). T1 and T2 denote 10% increase and decrease in threshold levels of miR-145 for inhibiting OCT4 (denoted by $\mu^0_{145 m_{O_c}}$). U1 and U2 denote 10% increase and decrease in threshold levels of miR-145 for inhibiting ZEB (denoted by $\mu^0_{145 m_Z}$). V1 and V2 denote 10% increase and decrease in threshold levels of ZEB for inhibiting miR-145 (denoted by $Z^0_{\mu_{145}}$). **(C) represents the cases with changes in the weight factors in the shifted Hill functions.** W1 and W2 denote increase and decrease in weight factor of ZEB inhibition on miR-200 (denoted by $\lambda_{Z,m_{200}}$) by 10%. X1 and X2 represent increase and decrease in weight factor of SNAIL inhibition on miR-200 (denoted by $\lambda_{S,m_{200}}$) by 10%. Y1 and Y2 denote respective increase and decrease in weight factor of SNAIL activation on ZEB (denoted by λ_{S,m_Z}). Z1 and Z2 denote the respective increase and decrease in weight factor of OCT4 inhibition of miR-145 (denoted by $\lambda_{O_c,\mu_{145}}$) by 10%. AA1 and AA2 denote increase and decrease in weight factor of inhibition

of OCT4 by miR-145 (denoted by $\lambda_{\mu_{145},m_{O_c}}$) by 10%. AB1 and AB2 denote increase and decrease in weight factor of ZEB self-activation (denoted by $/_{Z,m_Z}$) by 10%. AC1 and AC2 denote increase and decrease in weight factor of miR-145 inhibiting ZEB (denoted by λ_{μ_{145},m_Z}) by 10%. AD1 and AD2 denote increase and decrease in weight factor of ZEB inhibiting miR-145 (denoted by $\lambda_{Z,\mu_{145}}$) by 10%. AE1 and AE2 denote increase and decrease in the weight factor of activation of miR-200 by OCT4 (denoted by $\lambda_{O_c,\mu_{200}}$) by 10%.

Conversely, the parameter changes that are likely to decrease the effective ZEB levels that can induce EMT should enlarge the range of SNAIL levels for which the hybrid E/M state exists. This is indeed observed for cases such as when the production rate of ZEB mRNA is decreased (case B2), the degradation rate of ZEB mRNA is increase (case H1), the threshold ZEB levels for shifted Hill function of inhibition of ZEB on miR-200 is increased (case M1), the strength of ZEB activation by SNAIL is decreased (case Y2), the strength of ZEB self-activation is decreased (case AB2), and strength of inhibition of ZEB by miR-145 is increased (case AC2) (Figure S2).

Therefore, similar to the case of GRHL2, our prediction regarding the 'phenotypic stability factor' role of miR-145/OCT4 is quite robust to parameter variation, especially when ZEB levels are not too high. Also, the role of miR-145/OCT4 in increasing the association of E/M phenotype with stemness is most likely to be robust to most parameter changes, as long as let-7 levels are not too low, because similar to miR-200/ZEB, the LIN28/let-7 is sensitive towards the low levels of the microRNA, i.e. let-7 (44).

5. Levels of CDH1, VIM, ZEB1 and other PSFs in H2291, H1975, and H1299 cells

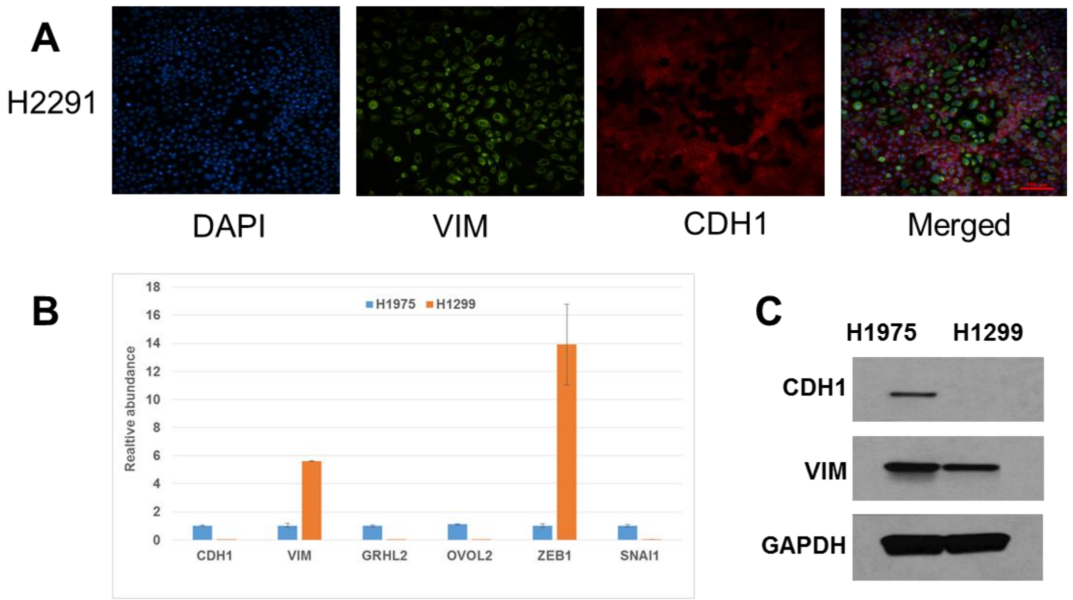

Figure S3: Levels of canonical epithelial and mesenchymal genes, EMT-inducing players and PSFs (A) Expression of CDH1 (E-cadherin) and VIM (Vimentin) in H2291 cells. Scale bar 100μm. **(B)** RT-PCR analysis of CDH1, VIM, OVOL2, GRHL2, ZEB1, and SNAI1 in H1975 (blue) and H1299 (orange). **(C)** Western-blot of H1975 and H1299 cells.

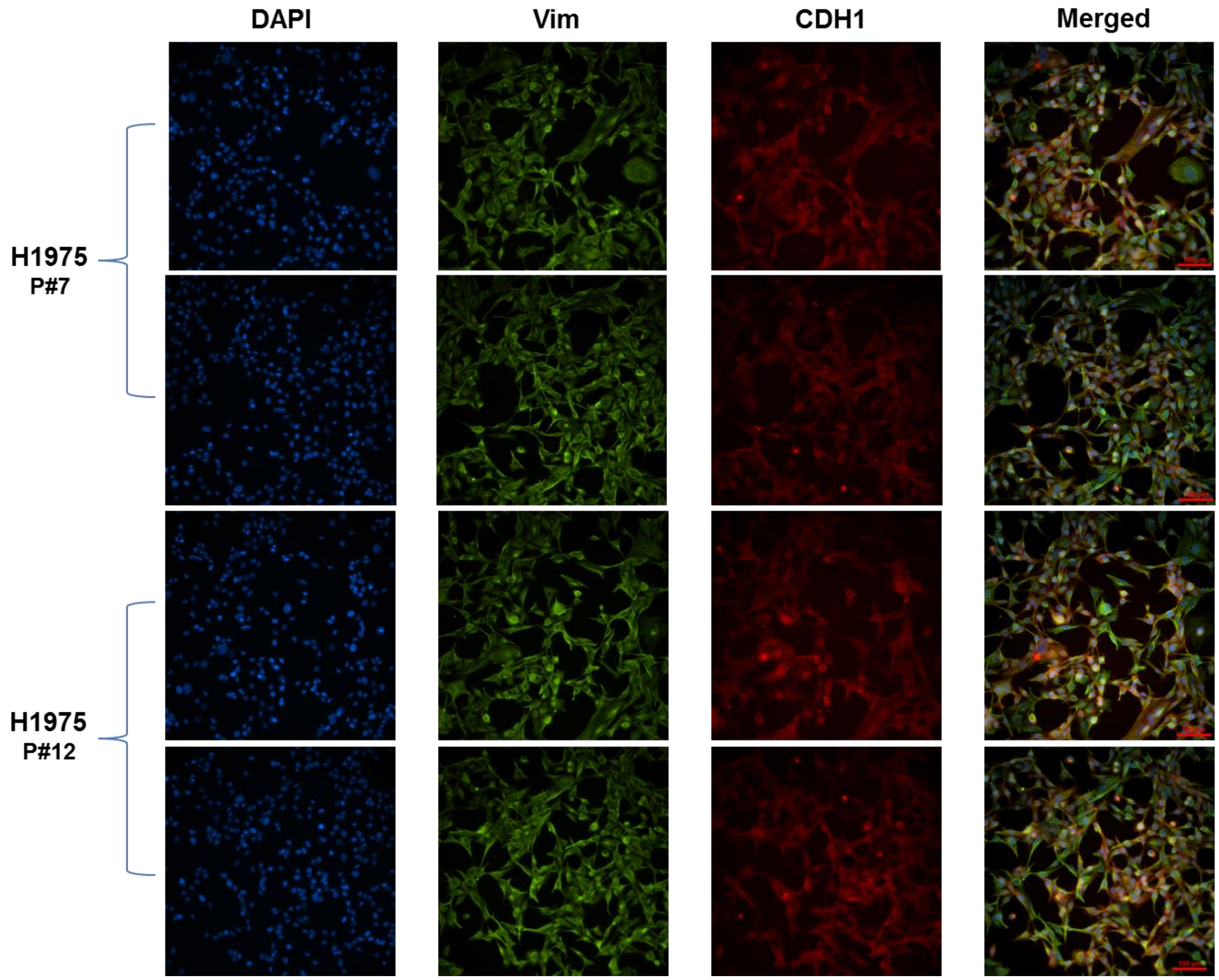

Figure S4: Expression of CDH1 (E-cadherin) and VIM (Vimentin) in different passages of the H1975 cells, as examined by immunofluorescence staining. Scale bar 100μm. P7 (passage #7) and P12 (passage #12) are separated by a period of two months.

6. Effect of external signal on miR-200/ZEB/GRHL2 circuit

An external activation signal on GRHL2 (SA) largely increases the area of the region corresponding to the epithelial phenotype - {E} and {E, E/M}; while an inhibition signal (SI) reduces this area and increases the area of the region corresponding to mesenchymal phenotype – {E, E/M, M} and {E/M, M} and {M} (Figure S5A, B), suggesting that GRHL2 knockdown can lead to a complete EMT, and that its overexpression can lead to MET. These simulations recapitulate experimental observations that ectopic

expression of GRHL2 can drive MET, while inhibiting it enables EMT (27,45). Similar results have been obtained for OVOL2 in earlier experimental and theoretical analysis (46–48). Further, activating miR-200 by p53 can drive MET (as reported (49)) both in presence and absence of GRHL2 (Figure S5C) that can behave as a 'phenotypic stability factor' for different values of p53 (Figure S5D).

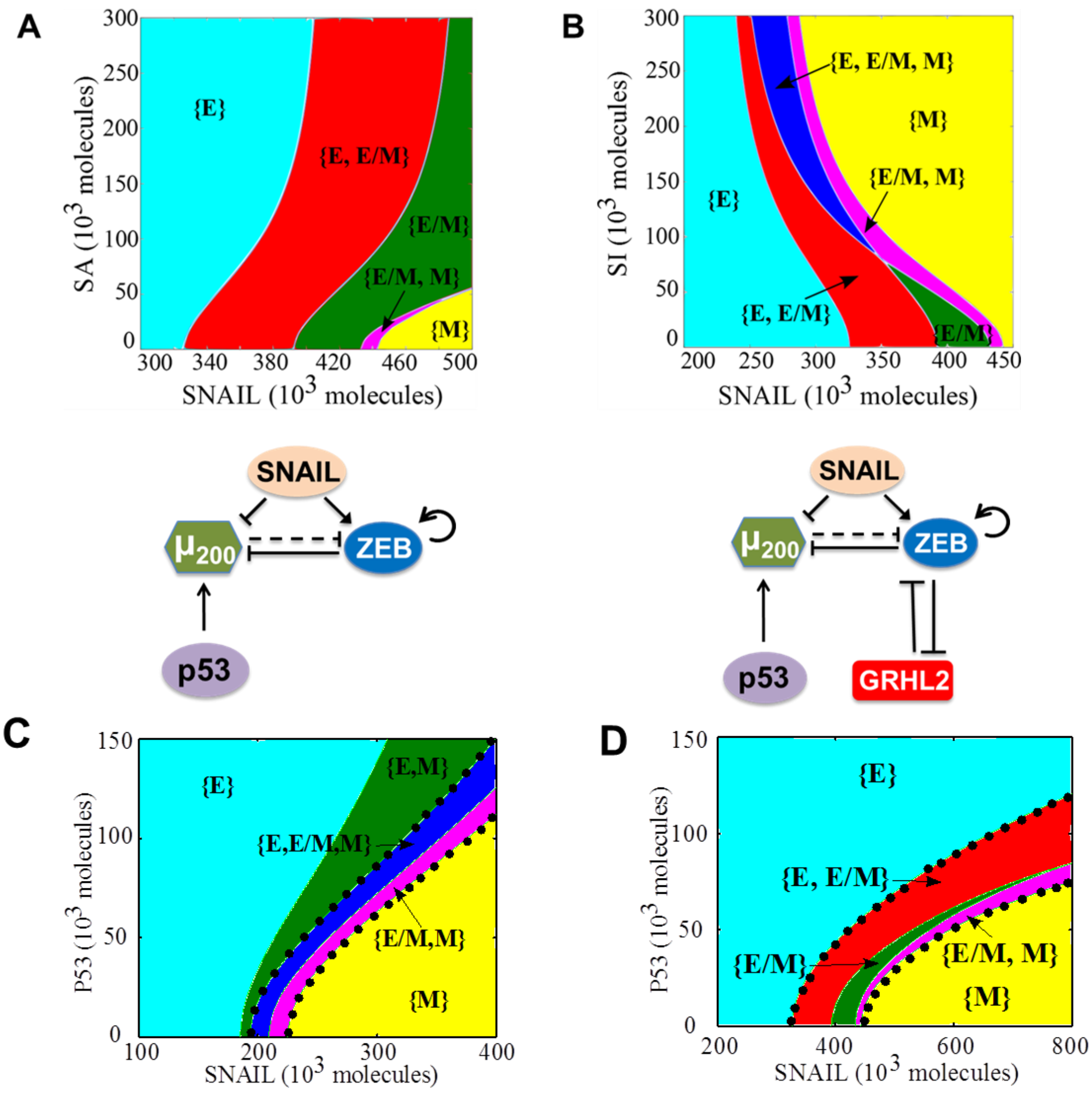

Figure S5: Phase diagram for miR-200/ZEB/GRHL2 circuit in response to SNAIL, external signals on GRHL2, and p53. **(A)** Response of the circuit to variable levels of SNAIL and an external activation signal on GRHL2 (SA). **(B)** shows the same as (A), but for an external inhibition signal on GRHL2 (SI). Different colors represent different phases, which represent the co-existence of possible stable states. **(C)** Response of the miR-200/ZEB circuit to variable levels of SNAIL and p53. **(D)** shows the same as (C), but for miR-200/ZEB/GRHL2 circuit. The area bounded by dotted black lines denotes the range of parameters for which the hybrid E/M state exists, either alone or in combination with other phenotypes. Different colored areas show different phases (co-existing phenotypes). Shifted Hill functions for these external signals are given in Table S1.

7. Knockdown of GRHL2 and OVOL2

Knockdown of GRHL2 and OVOL2 led to an increase in ZEB1 levels approximately 4-fold (Figure S6A) however, corresponding increase in SNAI1 was relatively less (Figure S6B), potentially because both GRHL2 and OVOL2 directly target ZEB1 and not SNAI1. Also, knockdown of GRHL2 tends to impact the proliferation for H1975 cells as they tend to undergo a complete EMT now. Unless otherwise specified, the figures (Figure 4, S6C,D) corresponding to GRHL2 and OVOL2 knockdown show the results for siGRHL#2 and siOVOL2#2.

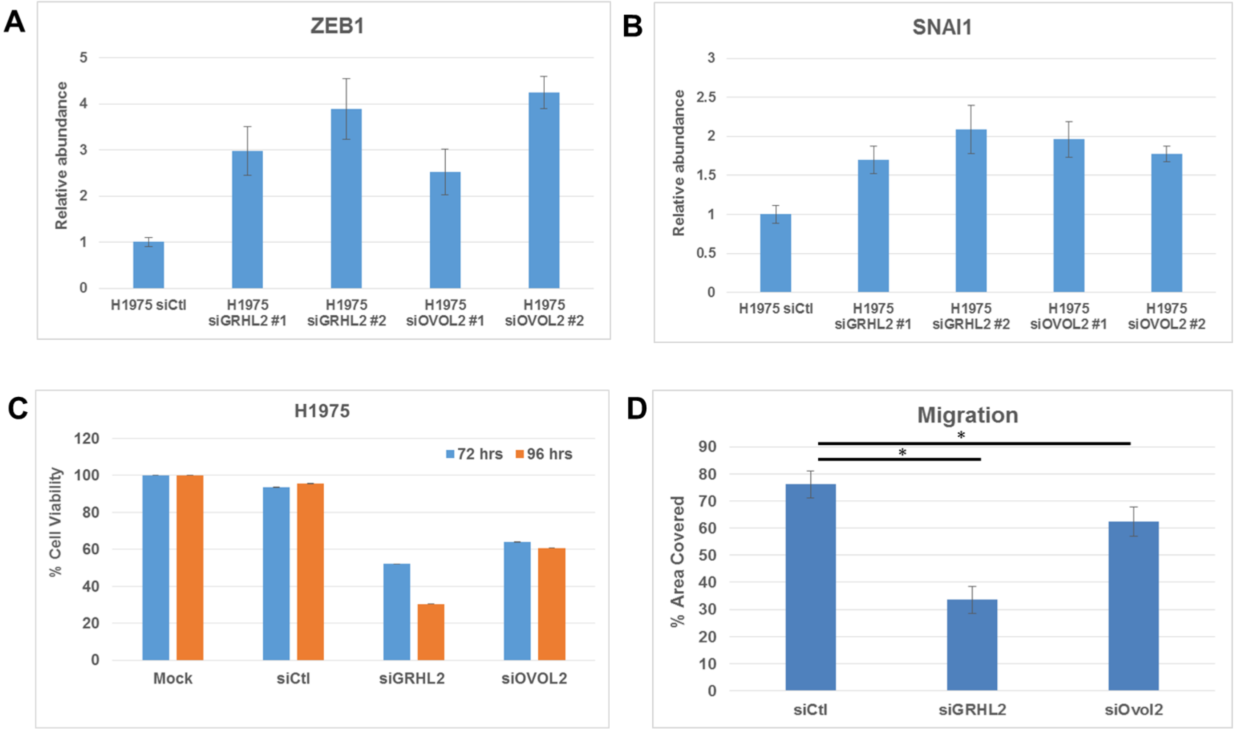

Figure S6: Characterizing the knockdown of GRHL2 and OVOL2 in H1975 cells. (A, B) RT-PCR analysis of ZEB1 and SNAI1 levels upon knockdown of GRHL2 and OVOL2. N=3 for each bar. **(C)** Cell viability at 72hours and 96 hours after treatment with siGRHL2 and siOVOL2. **(D)**Comparing the area covered during scratch assay of H1975, H1975-siOVOL2, H1975-siGRHL2 cells. *: $p<0.05$

8. Analysis of NCI 60 and PC3 clonal cell lines

The fold-change in levels of ZEB1, CDH1 and CDH3 suggest that OVOL2 might be a stronger driver of MET than OVOL1 in PC3 cells. Please note that care must be exercised in interpreting the values for OVOL1 in PC3-EMT-OVOL1 and that for OVOL2 in PC3-EMT-OVOL2 (marked by red in Figure S7A), because of the use of an overexpression vector. Nevertheless, OVOL2 being a strong driver was also reported in mammary development (47), and potentially explains why OVOL2-KO as compared to OVOL1-KO in H1975 cells is expected to yield a stronger phenotype.

ZEB1, OVOL2, CDH3, and GRHL2 have statistically significant different levels across the three phenotypes – E, M, and E/M – with the only exception being GRHL2 levels in E/M and M sets. This lack of significance can be attributed to tissue-specific differences as well as the presence of a few cell lines that have low (instead of medium) expression of CDH1 and VIM in the hybrid E/M cell line cluster (50).

A

Gene	Epi/EMT	EMT-OVOL2/EMT	EMT-OVOL1/EMT
ZEB1	0.02	0.24	0.57
CDH1	50.43	40.41	16.52
GRHL2	142.12	5.37	5.31
CDH3	16.02	17.79	8.94
OVOL2	25.89	2243.44	1.50
OVOL1	174.43	2.97	473.47

B

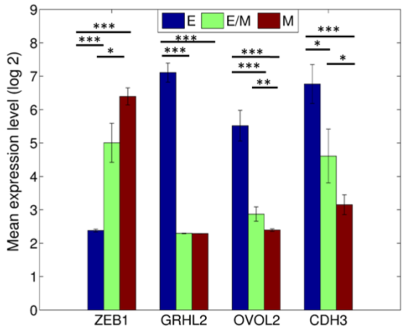

Figure S7: Analysis of NCI-60 panel and PC3-EMT-OVOL1, PC3-EMT-OVOL2. (A) Fold-change in the mean expression (log2) of OVOL2, OVOL1, ZEB1, CDH1, CDH3, GRHL2 in PC3-EMT-OVOL1 or 2, and PC3-Epi vs. PC3-EMT fold changes **(B)** Mean expression (log 2) of GRHL2, OVOL2, CDH3, and ZEB1 in different cell lines categorized as E, M and hybrid E/M. *, p<=0.05; **, p<0.005; ***, p<0.0005. Error bars represent standard error of mean.

9. GRHL2, OVOL2, and CDH3 levels may predict poor survival

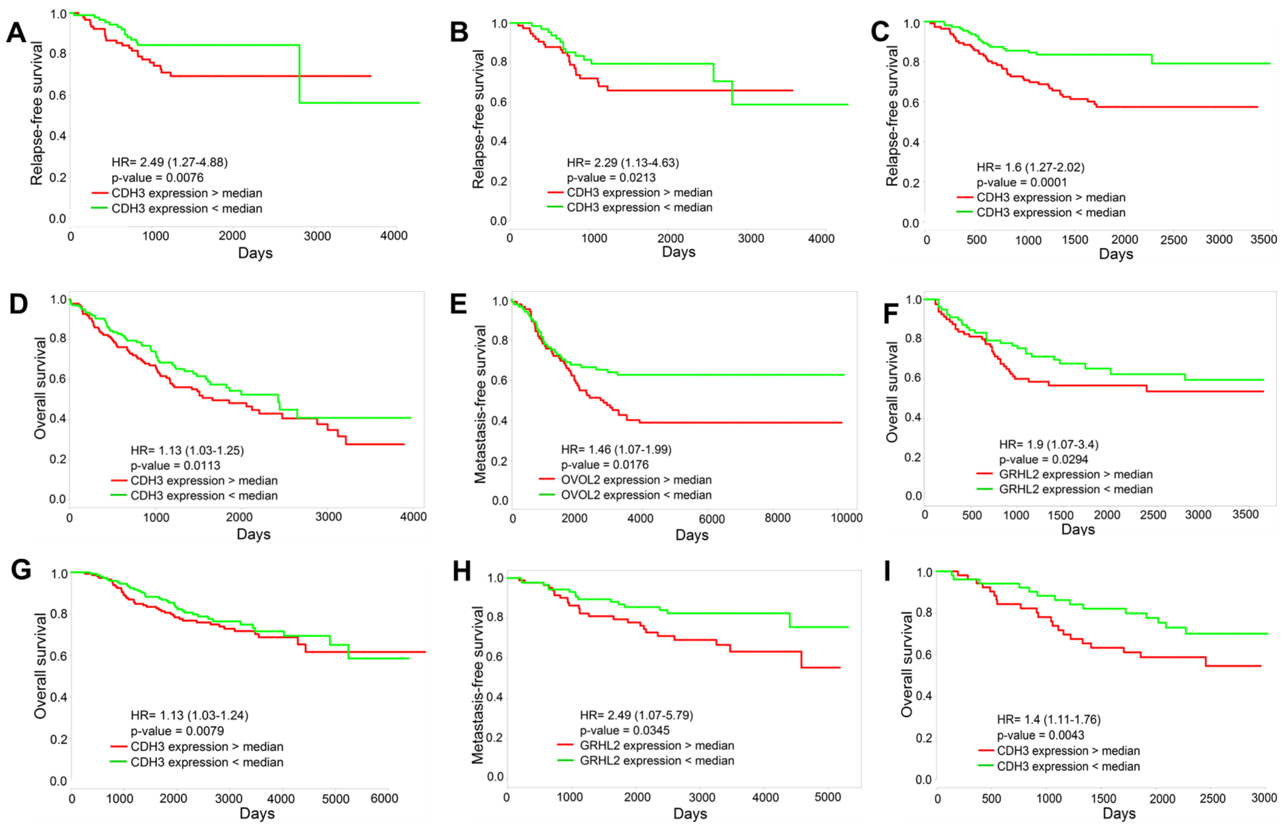

Figure S8: Survival analysis. Overall survival, relapse-free survival and metastasis-free survival for the expression of GRHL2, OVOL2 and CDH3 individually in multiple tissue types – **(A)** GSE14333 (n=187), **(B)** GSE17536 (n=135), **(C)** GSE31210 (n=225), **(D)** GSE41271 (n=274)**, (E)** GSE48408 (n=163), **(F)** GSE 24551 (n=159), **(G)** NKI (n=294), **(H)** GSE 6532_U133A (n=178), **(I)** GSE42568 (n=103).

We observed that in datasets where any one of the players – GRHL2, OVOL2, and CDH3 – correlates with poor survival, a combination of more than one of them also correlates with poor survival and usually has a lower p-value than the case with only one player. Also, we observed at least one case (GSE 3494 – n=235 patients) where the combined expression of GRHL2, OVOL2, and CDH3 correlated with poor overall survival in a statistical significant way, but none of them individually did (Table S4), indicating that looking at the combined expression might yield more predictive power. Looking at the combined expression might also help mitigate tissue-specific differences in expression levels of these players, each of which has been predicted to specifically associate with a hybrid E/M phenotype.

**Predictor variables**	**Hazard ratio**	**p-value**
GRHL2	1.25 (0.67-2.33)	0.4921
OVOL2	1.15 (0.58-2.29)	0.6942
CDH3	1.25 (0.96-1.62)	0.0935
GRHL2, OVOL2	1.4 (0.57-3.46)	0.4618
GRHL2, CDH3	1.51 (0.95-2.39)	0.0801
OVOL2, CDH3	1.77 (1-3.12)	0.0497
GRHL2, OVOL2, CDH3	2.11 (1-4.46)	0.0497

Table S4: Correlation of GRHL2, OVOL2, and CDH3 either individually or in combination with overall survival for GSE 3494.

10. Nullclines for the miR-200/ZEB/GRHL2 circuit

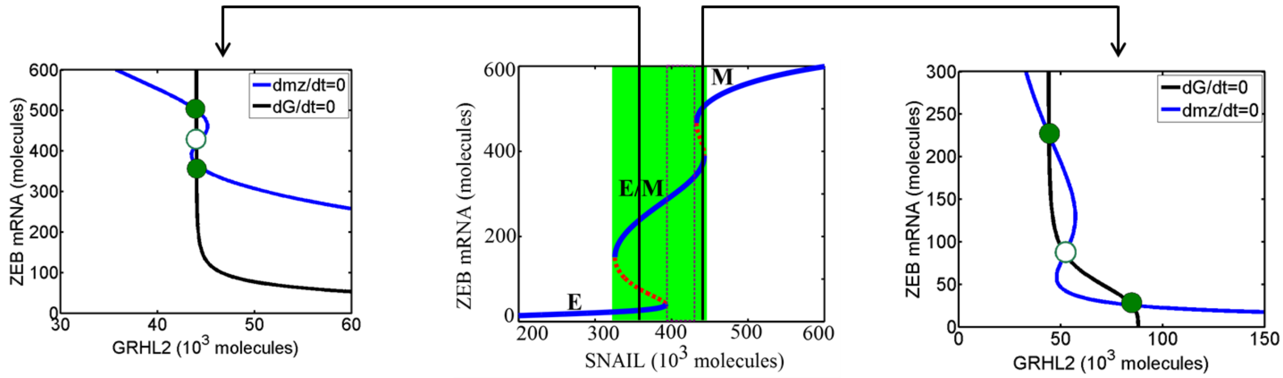

Figure S9: Nullclines and bifurcation of miR-200/ZEB/GRHL2 circuit. Middle panel shows the bifurcation of the circuit in response to SNAIL, and left and right panels represent nullclines of the circuit at two fixed values of SNAIL. Blue curve depicts the nullcline for the case when the system of equations given by (1)-(5) is set to zero, except equation (2). Blue curve depicts the nullcline for the case when the system of equations (1)-(5) is set to zero, except equation (5). Their intersections represent the different steady states of the circuit – the ones marked by green filled circles are stable, and the ones marked by hollow circles are unstable.

11. Coupling of miR-200/ZEB/GRHL2 with stemness module (LIN28/let-7)

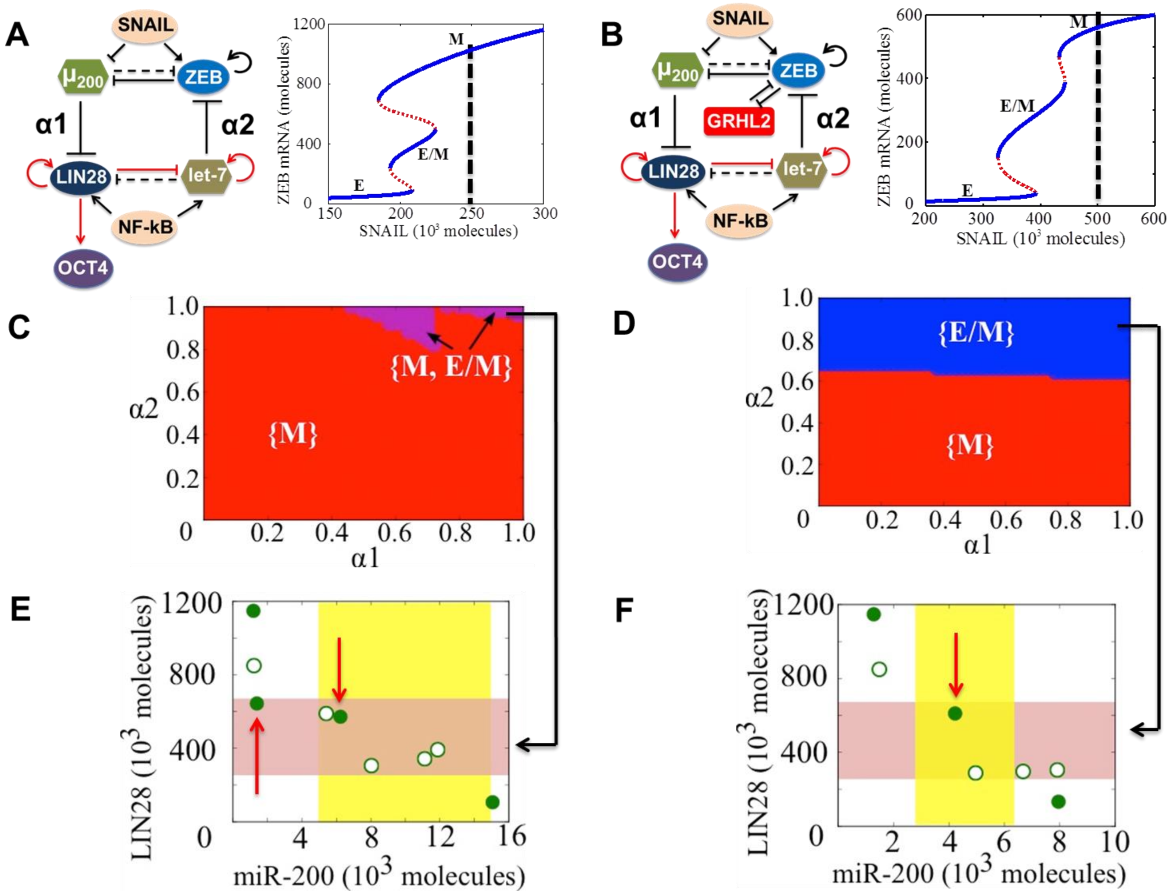

Figure S10 State-space characteristics of coupled networks miR-200/ZEB/LIN28/let-7 and miR-200/ZEB/LIN28/let-7/GRHL2, when cells are in {M} phase at α1=α2=0 (A, B) miR-200/ZEB/LIN28/let-7 and miR-200/ZEB/GRHL2/LIN28/let-7 circuits respectively. Black solid lines represent transcriptional regulation; red lines denote translational self-regulation of LIN28, and activation of its own processing by microRNA let-7, and dotted lines denote miRNA-mediated regulation. The parameters α1 and α2 denote the strength of the 'feed-forward coupling' (miR-200 inhibiting LIN28) and 'feed-backward coupling' (let-7 inhibiting ZEB) respectively, and lie between 0 and 1. Larger values denote stronger inhibition. The dashed line in the bifurcation diagrams next to the circuits shows the phase in which cells are present when there is no coupling between EMT and stemness circuits (α1=α2=0). Steady state diagram and the phase diagram in every column are for the circuit drawn in the topmost row of that column. **(C)** Phase diagram of the circuit miR-200/ZEB/LIN28/let-7 representing the values of (α1, α2) for which the different phenotypes can lie in stemness window, for SNAIL=250*10^3 molecules and NF-kB=25*10^3 molecules. **(D)** Phenotypic map of the coupled circuit at α1=α2=0.98 and at driving signals SNAIL=250*10^3 molecules and NF-kB=25*10^3 molecules. The red shaded area shows the 'stemness window' based on relative OCT4 levels, and the yellow shaded area represents the range of miR-200 levels for the existence of the hybrid E/M phenotype, as noted in (10) for (miR-200/ZEB) circuit and in SI section 10 for (miR-200/ZEB/GRHL2) circuit. **(E), (F)** represent a similar case for (C), (D) respectively but for the circuit with GRHL2, therefore SNAIL=500*10^3 molecules. Different colors represent different combinations of phenotypes that can gain stemness. The red arrows highlight the phenotypes that lie in

the ‘stemness window’. Green filled circles denote the stable steady states, and green hollow circles show the unstable steady states of the coupled circuits as denoted in (A) and (B).

12. Effective circuit for miR-200/ZEB/miR-145/OCT4

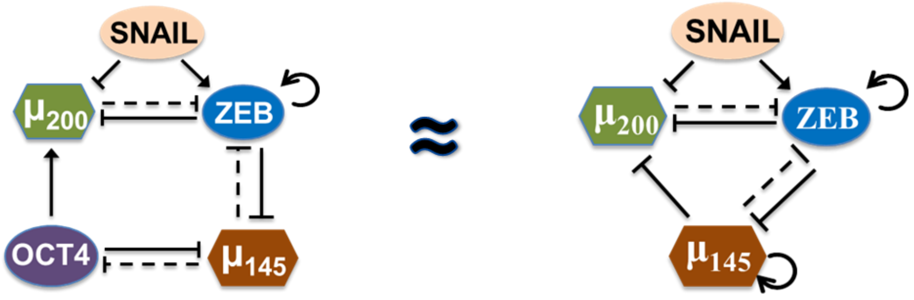

Figure S11: The miR-200/ZEB circuit coupled with miR-145/OCT4, and the reduced or effective miR-200/ZEB/miR-145 circuit, where miR-145 both inhibits miR-200 and indirectly self-activates via OCT4.

The mutual inhibition between OCT4 and miR-145 can be considered to a self-activation due to the action of miR-145, i.e. inhibiting its own inhibitor. Furthermore, the inhibition of miR-200 by miR-145 via OCT4 can therefore be replaced with a direct inhibitory link. The inhibition of miR-145 on ZEB is expected to be relatively stronger because in addition to directly inhibiting ZEB, miR-145 can also inhibit the nuclear translocation of β-catenin that can fuel the completion of ZEB by activating the TCF4 complex that can further activate ZEB and lead to more release of membranous β-catenin. Nonetheless, this reduction into the effective circuit is likely to depend on relative strengths of the links in the network.

13. Nullclines for the I/miR-200/ZEB circuit.

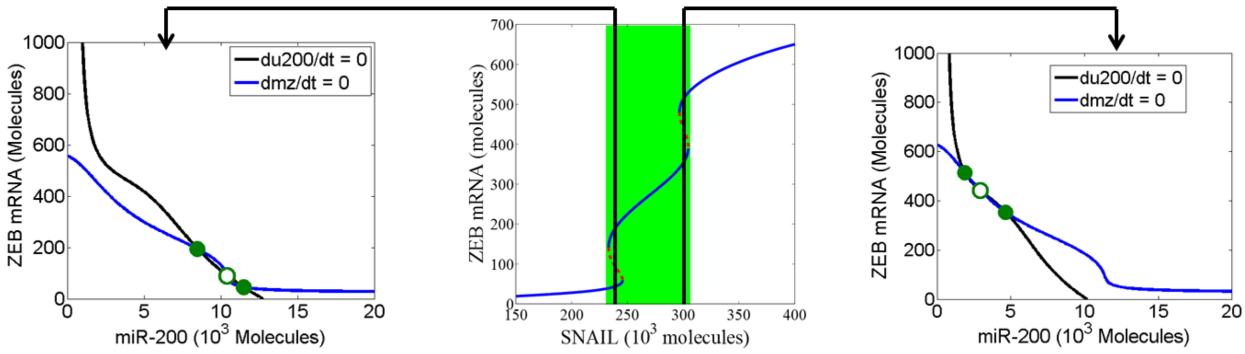

Figure S12: Nullclines and bifurcation of I/miR-200/ZEB circuit. The middle panel shows the bifurcation of the circuit in response to SNAIL. The left and right panels represent nullclines of the circuit at two fixed values of SNAIL. Red curve depicts the nullcline for the case when the system of equations (1), (21-22) is set to zero, except equation (1). The other curve depicts the nullcline for the case when all these equations are set to zero, except equation (22). Their intersections represent the different steady states of the circuit – green filled circles denote stable ones, and the ones marked by hollow circles are unstable.

14. Coupling of I/miR-200/ZEB with stemness module (LIN28/let-7)

First, we calculate the association of different phenotypes with stemness – both in the presence of incoherent signal I and its absence – when cells are in {M} phase at α1= α2=0 (Figure S13 A,B). The incoherent signal I enables a relatively much larger parameter space (α1, α2) for the association of the hybrid E/M phenotype with stemness (Figure S13 C, D). Specifically, at strong bidirectional coupling between the EMT and stemness modules, only the hybrid E/M phenotypes gains stemness, and the M phenotype is pushed out of the 'stemness window' in presence of I (compare phenotypes in the red area in Figure S13 E,F).

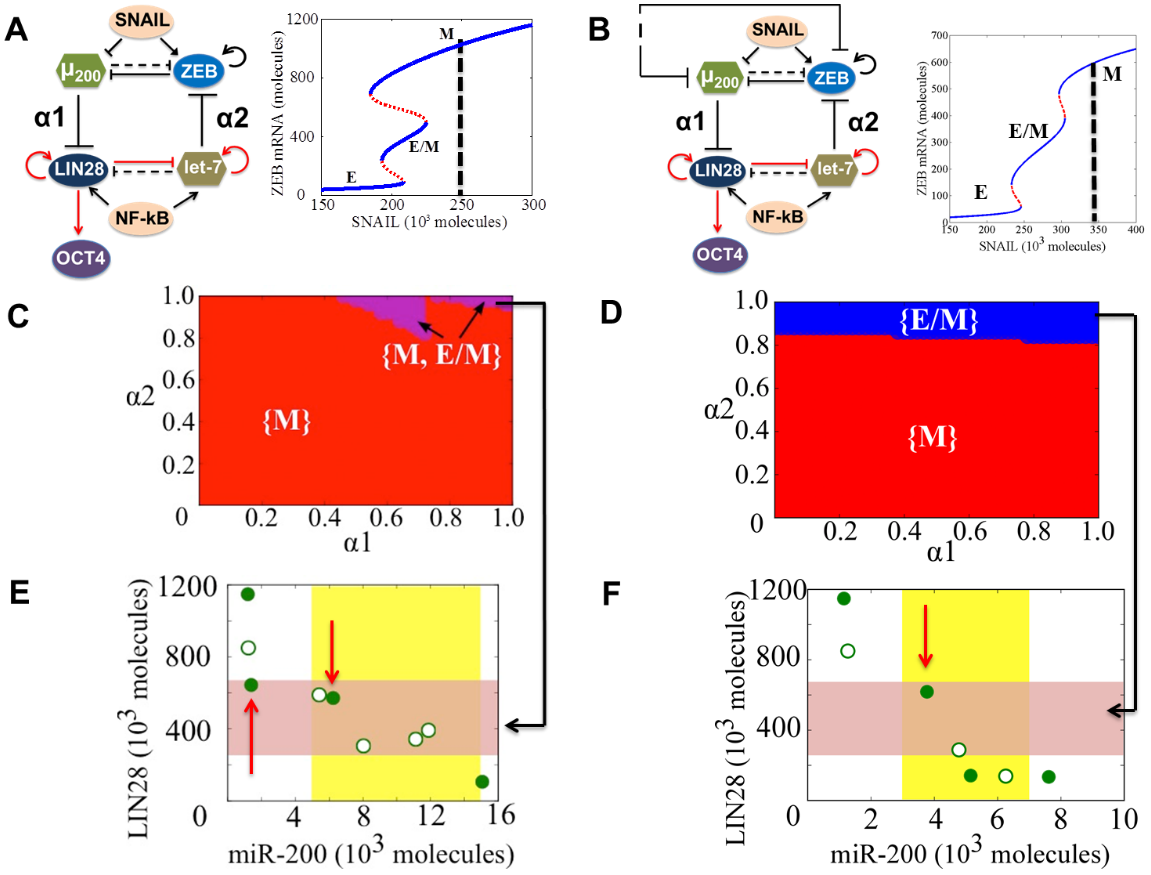

Figure S13: State-space characteristics of coupled networks miR-200/ZEB/LIN28/let-7 and I/miR-200/ZEB/LIN28/let-7, when cells are in {M} phase at α1=α2=0 (A, B) miR-200/ZEB/LIN28/let-7 and I/ miR-200/ZEB/LIN28/let-7 circuits respectively. Black solid lines represent transcriptional regulation; red lines denote translational self-regulation of LIN28, and activation of its own processing by microRNA let-7, and dotted lines denote miRNA-mediated regulation. The parameters α1 and α2 denote the strength of the 'feed-forward coupling' (miR-200 inhibiting LIN28) and 'feed-backward coupling' (let-7 inhibiting ZEB) respectively, and lie between 0 and 1. Larger values denote stronger inhibition. The dashed line in the bifurcation diagrams next to the circuits shows the phase in which cells are present when there is no coupling between EMT and stemness circuits (α1=α2=0). Steady state diagram and the

phase diagram in every column are for the circuit drawn in the topmost row of that column. **(C)** Phase diagram of the circuit I/ miR-200/ZEB/LIN28/let-7 representing the values of (α1, α2) for which the different phenotypes can lie in stemness window, for SNAIL=250*10^3 molecules and NF-kB=25*10^3 molecules. **(D)** Phenotypic map of the coupled circuit at α1=α2=0.98 and at driving signals SNAIL=250*10^3 molecules and NF-kB=25*10^3 molecules. The red shaded area shows the 'stemness window' based on relative OCT4 levels, and the yellow shaded area represents the range of miR-200 levels for the existence of the hybrid E/M phenotype, as noted in (10) for (miR-200/ZEB) circuit and in SI section 13 for the (I/miR-200/ZEB) circuit. **(E), (F)** represent a similar case for (C), (D) respectively but for the circuit after incorporating I (the incoherent external signal on (miR-200/ZEB)), hence SNAIL =350*10^3 molecules. Different colors represent different combinations of phenotypes that can gain stemness. The red arrows highlight the phenotypes that lie in the 'stemness window'. Green filled circles denote the stable steady states, and green hollow circles show the unstable steady states of the coupled circuits as denoted in (A) and (B).

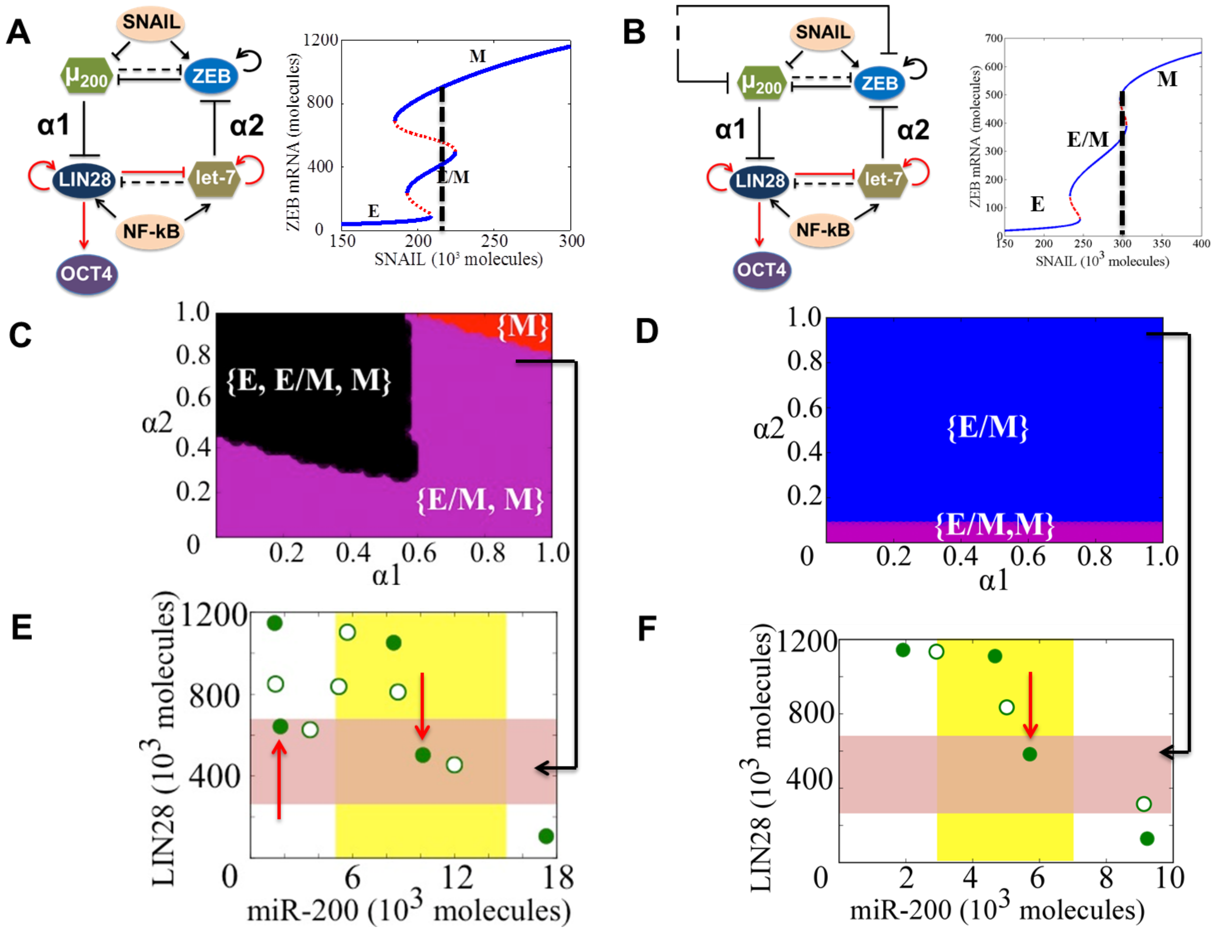

Figure S14: State-space characteristics of coupled networks miR-200/ZEB/LIN28/let-7 and I/miR-200/ZEB/LIN28/let-7, when cells are in {E/M, M} phase at α1=α2=0 (A, B) miR-200/ZEB/LIN28/let-7 and I/ miR-200/ZEB/LIN28/let-7 circuits respectively. Black solid lines represent transcriptional regulation; red lines denote translational self-regulation of LIN28, and activation of its own processing by

microRNA let-7, and dotted lines denote miRNA-mediated regulation. The parameters α1 and α2 denote the strength of the 'feed-forward coupling' (miR-200 inhibiting LIN28) and 'feed-backward coupling' (let-7 inhibiting ZEB) respectively, and lie between 0 and 1. Larger values denote stronger inhibition. The dashed line in the bifurcation diagrams next to the circuits shows the phase in which cells are present when there is no coupling between EMT and stemness circuits (α1=α2=0). Steady state diagram and the phase diagram in every column are for the circuit drawn in the topmost row of that column. **(C)** Phase diagram of the circuit I/ miR-200/ZEB/LIN28/let-7 representing the values of (α1, α2) for which the different phenotypes can lie in the 'stemness window', for SNAIL=220*10^3 molecules and NF-kB=25*10^3 molecules. **(D)** Phenotypic map of the coupled circuit at α1=α2=0.98 and at driving signals SNAIL=220*10^3 molecules and NF-kB=25*10^3 molecules. The red shaded area shows the 'stemness window' based on relative OCT4 levels, and the yellow shaded area represents the range of miR-200 levels for the existence of the hybrid E/M phenotype, as noted in (10) for (miR-200/ZEB) circuit and in SI section 12 for the (I/miR-200/ZEB) circuit. **(E), (F)** represent a similar case for (C), (D) respectively but for the circuit after incorporating I (the incoherent external signal on (miR-200/ZEB)), hence SNAIL =300*10^3 molecules. Different colors represent different combinations of phenotypes that can gain stemness. Red arrows denote the phenotypes that lie in the 'stemness window'. Green filled circles denote the stable steady states, and green hollow circles show the unstable steady states of the coupled circuits as denoted in (A) and (B).

Second, we calculate the association of different phenotypes with stemness – both in the presence of incoherent signal I and its absence – when cells are in {E/M, M} phase at α1= α2=0 (Figure S14 A,B). Similar to the previous results, the incoherent signal I enable a relatively much larger parameter space (α1, α2) for the association of the hybrid E/M phenotype with stemness (Figure S14 C, D). Again, consistently, at strong bidirectional coupling between the EMT and stemness modules, only the hybrid E/M phenotypes gains stemness, and the M phenotype is pushed out of the 'stemness window' in the presence of I (compare phenotypes in the red area in Figure S14 E,F; M and E phenotypes can still exist but not lie in the 'stemness window'), thereby denoting that the incoherent signal I associates the hybrid E/M phenotype with stemness largely independent of initial conditions in the simulation.

14. Bifurcations for coupling of miR-200/ZEB in different topologies

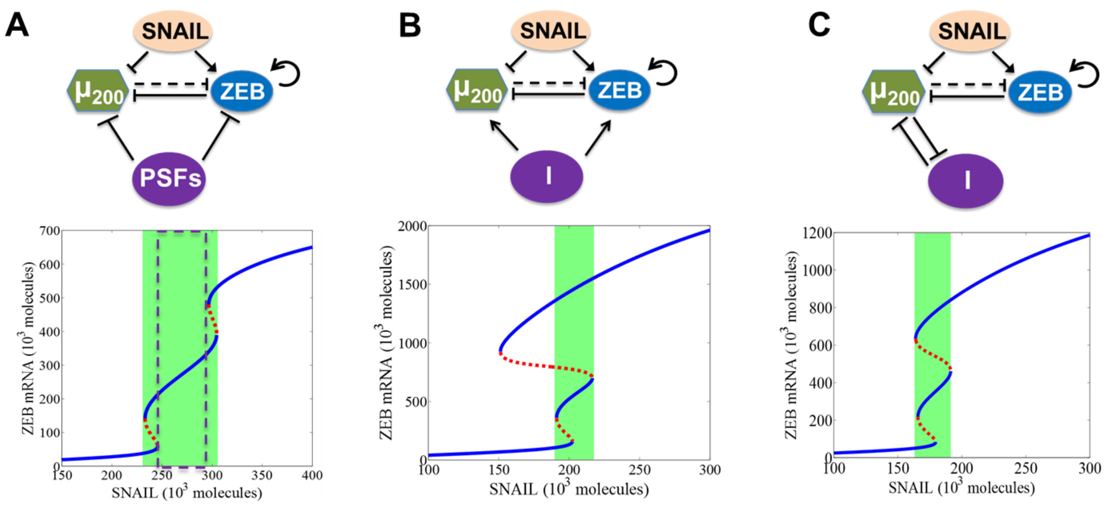

Figure S15: Bifurcations for coupling of miR-200/ZEB in different topologies. (A) An external incoherent signal that inhibits both miR-200 and ZEB. (B) An external incoherent signal that activates both miR-200 and ZEB. (C) A double negative feedback loop with miR-200. Bifurcation diagrams below correspond to the behavior of the circuits just above. The green shaded region represents the range of SNAIL levels for which the hybrid E/M phenotype exists either alone or in combination with other phenotypes. The dotted rectangle denotes the range of SNAIL levels for which hybrid the E/M phenotype can exist alone. Blue solid lines represent stable steady states (phenotypes), and red dashed lines show unstable steady states.

References

1. Bracken CP, Gregory PA, Kolesnikoff N, Bert AG, Wang J, Shannon MF, Goodall GJ. A double-negative feedback loop between ZEB1-SIP1 and the microRNA-200 family regulates epithelial-mesenchymal transition. Cancer Res. 2008;68(19):7846–54.
2. Burk U, Schubert J, Wellner U, Schmalhofer O, Vincan E, Spaderna S, Brabletz T. A reciprocal repression between ZEB1 and members of the miR-200 family promotes EMT and invasion in cancer cells. EMBO Rep. 2008;9:582–9.
3. Siemens H, Jackstadt R, Hünten S, Kaller M, Menssen A, Götz U, Hermeking H. miR-34 and SNAIL form a double-negative feedback loop to regulate epithelial-mesenchymal transitions. Cell Cycle. 2011. p. 4256–71.
4. Lu M, Jolly MK, Levine H, Onuchic JN, Ben-Jacob E. MicroRNA-based regulation of epithelial-hybrid-mesenchymal fate determination. Proc Natl Acad Sci. 2013 Oct;110(45):18144–9.
5. Brabletz S, Brabletz T. The ZEB/miR-200 feedback loop--a motor of cellular plasticity in development and cancer? EMBO Rep. 2010;11(9):670–7.
6. Hurteau GJ, Carlson JA, Roos E, Brock GJ. Stable expression of miR-200c alone is sufficient to regulate TCF8 (ZEB1) and restore E-cadherin expression. Cell Cycle. 2009;8:2064–9.
7. Hill L, Browne G, Tulchinsky E. ZEB/miR-200 feedback loop: at the crossroads of signal

transduction in cancer. Int J Cancer [Internet]. 2013 Feb 15 [cited 2013 Nov 17];132(4):745–54. Available from: http://www.ncbi.nlm.nih.gov/pubmed/22753312

8. Preca B-T, Bajdak K, Mock K, Sundararajan V, Pfannstiel J, Maurer J, Wellner U, Hopt UT, Brummer T, Brabletz S, Brabletz T, Stemmler MP. A self-reinforcing CD44s/ZEB1 feedback loop maintains EMT and stemness properties in cancer cells. Int J Cancer. 2015;

9. Guaita S, Puig I, Franci C, Garrido M, Dominguez D, Batlle E, Sancho E, Dedhar S, De Herreros AGG, Baulida J. Snail induction of epithelial to mesenchymal transition in tumor cells is accompanied by MUC1 repression and ZEB1 expression. J Biol Chem. 2002;277(42):39209–16.

10. Lu M, Jolly MK, Levine H, Onuchic JN, Ben-Jacob E. MicroRNA-based regulation of epithelial-hybrid-mesenchymal fate determination. Proc Natl Acad Sci U S A. 2013 Oct;110(45):18174–9.

11. Kim T, Veronese A, Pichiorri F, Lee TJ, Jeon Y-J, Volinia S, Pineau P, Marchio A, Palatini J, Suh S-S, Alder H, Liu C-G, Dejean A, et al. p53 regulates epithelial-mesenchymal transition through microRNAs targeting ZEB1 and ZEB2. J Exp Med. 2011 May 9;208(5):875–83.

12. Yang X, Lin X, Zhong X, Kaur S, Li N, Liang S, Lassus H, Wang L, Katsaros D, Montone K, Zhao X, Zhang Y, Bützow R, et al. Double-negative feedback loop between reprogramming factor LIN28 and microRNA let-7 regulates aldehyde dehydrogenase 1-positive cancer stem cells. Cancer Res. 2010;70:9463–72.

13. Piskounova E, Polytarchou C, Thornton JE, Lapierre RJ, Pothoulakis C, Hagan JP, Iliopoulos D, Gregory RI. Lin28A and Lin28B inhibit let-7 MicroRNA biogenesis by distinct mechanisms. Cell. .; 2011;147(5):1066–79.

14. Hafner M, Max KE a, Bandaru P, Morozov P, Gerstberger S, Brown M, Molina H, Tuschl T. Identification of mRNAs bound and regulated by human LIN28 proteins and molecular requirements for RNA recognition. RNA. 2013;19(5):613–26.

15. Zisoulis DG, Kai ZS, Chang RK, Pasquinelli AE. Autoregulation of microRNA biogenesis by let-7 and Argonaute. Nature. 2012;486(7404):541–4.

16. Jolly MK, Huang B, Lu M, Mani SA, Levine H, Ben-Jacob E. Towards elucidating the connection between epithelial − mesenchymal transitions and stemness. J R Soc Interface. 2014;11(101):20140962.

17. Qiu C, Ma Y, Wang J, Peng S, Huang Y. Lin28-mediated post-transcriptional regulation of Oct4 expression in human embryonic stem cells. Nucleic Acids Res. 2010;38(4):1240–8.

18. Niwa H, Miyazaki J, Smith AG. Quantitative expression of Oct-3/4 defines differentiation, dedifferentiation or self-renewal of ES cells. Nat Genet. 2000;24(4):372–6.

19. Theunissen TW, Van Oosten AL, Castelo-Branco G, Hall J, Smith A, Silva JCR. Nanog overcomes reprogramming barriers and induces pluripotency in minimal conditions. Curr Biol. 2011;21(1):65–71.

20. Karwacki-Neisius V, Göke J, Osorno R, Halbritter F, Ng JH, Weiße AY, Wong FCK, Gagliardi A, Mullin NP, Festuccia N, Colby D, Tomlinson SR, Ng HH, et al. Reduced Oct4 expression directs a robust pluripotent state with distinct signaling activity and increased enhancer occupancy by Oct4 and Nanog. Cell Stem Cell. 2013;12(5):531–45.

21. Shu J, Wu C, Wu Y, Li Z, Shao S, Zhao W, Tang X, Yang H, Shen L, Zuo X, Yang W, Shi Y, Chi X, et al. Induction of pluripotency in mouse somatic cells with lineage specifiers. Cell. Elsevier Inc.; 2013;153(5):963–75.

22. Kong D, Banerjee S, Ahmad A, Li Y, Wang Z, Sethi S, Sarkar FH. Epithelial to mesenchymal transition is mechanistically linked with stem cell signatures in prostate cancer cells. PLoS One. 2010 Jan;5(8):e12445.

23. Yong SL, Dutta A. The tumor suppressor microRNA let-7 represses the HMGA2 oncogene. Genes Dev. 2007;21(9):1025–30.

24. Guo L, Chen C, Shi M, Wang F, Chen X, Diao D, Hu M, Yu M, Qian L, Guo N. Stat3-coordinated Lin-28–let-7–HMGA2 and miR-200–ZEB1 circuits initiate and maintain oncostatin M-driven epithelial–mesenchymal transition. Oncogene. 2013;32(45):5272–82.

25. Tan EJ, Thuault S, Caja L, Carletti T, Heldin CH, Moustakas A. Regulation of transcription factor twist expression by the DNA architectural protein high mobility group A2 during epithelial-to-mesenchymal transition. J Biol Chem. 2012;287(10):7134–45.

26. Thuault S, Valcourt U, Petersen M, Manfioletti G, Heldin CH, Moustakas A. Transforming growth factor-β employs HMGA2 to elicit epithelial-mesenchymal transition. J Cell Biol. 2006;174(2):175–83.

27. Varma S, Cao Y, Tagne JB, Lakshminarayanan M, Li J, Friedman TB, Morell RJ, Warburton D, Kotton DN, Ramirez MI. The transcription factors grainyhead-like 2 and NK2-homeobox 1 form a regulatory loop that coordinates lung epithelial cell morphogenesis and differentiation. J Biol Chem. 2012;287(44):37282–95.

28. Cieply B, Riley IV P, Pifer PM, Widmeyer J, Addison JB, Ivanov A V., Denvir J, Frisch SM. Suppression of the epithelial-mesenchymal transition by grainyhead-like-2. Cancer Res. 2012;72(9):2440–53.

29. Cieply B, Farris J, Denvir J, Ford HL, Frisch SM. Epithelial-Mesenchymal Transition and Tumor Suppression Are Controlled by a Reciprocal Feedback Loop between ZEB1 and Grainyhead-like-2. Cancer Res. 2013;73:6299–309.

30. Xu N, Papagiannakopoulos T, Pan G, Thomson J a., Kosik KS. MicroRNA-145 Regulates OCT4, SOX2, and KLF4 and Represses Pluripotency in Human Embryonic Stem Cells. Cell. 2009;137(4):647–58.

31. Wang G, Guo X, Hong W, Liu Q, Wei T, Lu C, Gao L, Ye D, Zhou Y. Critical regulation of miR-200 / ZEB2 pathway in Oct4 / Sox2-induced mesenchymal-to-epithelial transition and induced pluripotent stem cell generation. Proc Natl Acad Sci U S A. 2013;110(8):2858–63.

32. Lewis BP, Burge CB, Bartel DP. Conserved seed pairing, often flanked by adenosines, indicates that thousands of human genes are microRNA targets. Cell. 2005;120(1):15–20.

33. Polytarchou C, Iliopoulos D, Struhl K. An integrated transcriptional regulatory circuit that reinforces the breast cancer stem cell state. Proc Natl Acad Sci U S A. 2012;109(36):14470–5.

34. Sachdeva M, Zhu S, Wu F, Wu H, Walia V, Kumar S, Elble R, Watabe K, Mo Y-Y. p53 represses c-Myc through induction of the tumor suppressor miR-145. Proc Natl Acad Sci U S A. 2009;106(9):3207–12.

35. Desjardins A, Yang A, Bouvette J, Omichinski JG, Legault P. Importance of the NCp7-like domain in the recognition of pre-let-7g by the pluripotency factor Lin28. Nucleic Acids Res. 2012;40(4):1767–77.

36. Milo R, Jorgensen P, Moran U, Weber G, Springer M. BioNumbers--the database of key numbers in molecular and cell biology. Nucleic Acids Res. 2010 Jan;38(Database issue):D750–3.

37. Schwanhäusser B, Busse D, Li N, Dittmar G, Schuchhardt J, Wolf J, Chen W, Selbach M. Global quantification of mammalian gene expression control. Nature. 2011 May;473(7347):337–42.

38. Lim LP, Lau NC, Weinstein EG, Abdelhakim A, Yekta S, Rhoades MW, Burge CB, Bartel DP. The microRNAs of Caenorhabditis elegans. Genes Dev. 2003 Apr;17(8):991–1008.

39. Eden E, Geva-Zatorsky N, Issaeva I, Cohen A, Dekel E, Danon T, Cohen L, Mayo A, Alon U. Proteome Half-Life Dynamics in Living Human Cells. Science (80-). 2011 Feb;331(6018):764–8.

40. Yang E, van Nimwegen E, Zavolan M, Rajewsky N, Schroeder M, Magnasco M, Darnell JE. Decay rates of human mRNAs: correlation with functional characteristics and sequence attributes. Genome Res. 2003 Aug;13(8):1863–72.

41. Gantier MP, McCoy CE, Rusinova I, Saulep D, Wang D, Xu D, Irving AT, Behlke MA, Hertzog PJ, Mackay F, Williams BRG. Analysis of microRNA turnover in mammalian cells following Dicer1 ablation. Nucleic Acids Res. 2011 Jul;39(13):5692–703.

42. Khanin R, Vinciotti V. Computational Modeling of Post-Transcriptional Gene Regulation by MicroRNAs. J Comput Biol. 2008 Apr;15(3):305–16.

43. Jolly MK, Jia D, Boareto M, Mani SA, Pienta KJ, Ben-Jacob E, Levine H. Coupling the modules of EMT and stemness : A tunable "stemness window" model. Oncotarget. 2015;6(28):25161–74.

44. Jolly MK, Huang B, Lu M, Mani SA, Levine H, Ben-Jacob E. Towards elucidating the connection between epithelial–mesenchymal transitions and stemness. J R Soc Interface. 2014 Dec;11(101):20140962.

45. Werner S, Frey S, Riethdorf S, Schulze C, Alawi M, Kling L, Vafaizadeh V, Sauter G, Terracciano L, Schumacher U, Pantel K, Assmann V. Dual roles of the transcription factor grainyhead-like 2 (GRHL2) in breast cancer. J Biol Chem. 2013;288(32):22993–3008.

46. Roca H, Hernandez J, Weidner S, McEachin RC, Fuller D, Sud S, Schumann T, Wilkinson JE, Zaslavsky A, Li H, Maher CA, Daignault-Newton S, Healy PN, et al. Transcription Factors OVOL1 and OVOL2 Induce the Mesenchymal to Epithelial Transition in Human Cancer. PLoS One. 2013;8:e76773.

47. Watanabe K, Villarreal-Ponce A, Sun P, Salmans ML, Fallahi M, Andersen B, Dai X. Mammary morphogenesis and regeneration require the inhibition of EMT at terminal end buds by Ovol2 transcriptional repressor. Dev Cell. 2014;29(1):59–74.

48. Jia D, Jolly MK, Boareto M, Parsana P, Mooney SM, Pienta KJ, Levine H, Ben-Jacob E. OVOL guides the epithelial-hybrid-mesenchymal transition. Oncotarget. 2015;6(17):15436–48.

49. Chang C-J, Chao C-H, Xia W, Yang J-Y, Xiong Y, Li C-W, Yu W-H, Rehman SK, Hsu JL, Lee H-H, Liu M, Chen C-T, Yu D, et al. p53 regulates epithelial-mesenchymal transition and stem cell properties through modulating miRNAs. Nat Cell Biol. 2011;13(3):317–23.

50. Park S-MM, Gaur AB, Lengyel E, Peter ME. The miR-200 family determines the epithelial phenotype of cancer cells by targeting the E-cadherin repressors ZEB1 and ZEB2. Genes Dev. 2008 Apr 1;22(7):894–907.
